# Virtual reality-based inhibition training influences food-related responses: no additional effects of repetitive transcranial magnetic stimulation

**DOI:** 10.3389/fpsyt.2026.1801985

**Published:** 2026-05-08

**Authors:** Hyeong Ha Kim, HeungSik Yoon, Sang Hee Kim

**Affiliations:** 1 Affective Cognition Laboratory, Department of Brain and Cognitive Engineering, Korea University, Seoul, Republic of Korea; 2 Institute of Brain and Cognitive Engineering, Korea University, Seoul, Republic of Korea

**Keywords:** food choice, food craving, implicit attitudes, no-go inhibition training, repetitive transcranial magnetic stimulation (rTMS), virtual reality (VR)

## Abstract

Combining cognitive inhibition training with brain stimulation techniques has received increasing attention as a potential approach to modulating maladaptive food craving and food intake. Building on previous work in this line of research, the current study examined whether virtual reality (VR)-based no-go inhibition training paired with repetitive transcranial magnetic stimulation (rTMS) modulates implicit food-related attitudes, craving and food-choice behaviors. Healthy women with high trait food cravings and a preference for high-calorie foods were assigned to one of four groups in a 2 (rTMS: active vs. sham) × 2 (training: no-go vs. neutral) between-subjects design. High-frequency rTMS was applied over the left dorsolateral prefrontal cortex (DLPFC), and no-go training was implemented in a VR environment using food stimuli tailored to participants’ self-reported preferences. Implicit attitudes and food craving were assessed before and after the intervention, while food choice was measured post-intervention only. Following training, the no-go group showed reduced positive implicit attitudes toward high-calorie foods and increased craving for low-calorie foods compared to pre-training levels, whereas no such changes were observed in the neutral group. Moreover, compared to the neutral group, the no-go group made healthier food choices. No-go training effects on food choice were more pronounced among individuals with low-to-moderate baseline preferences for high-calorie foods. In contrast, no significant main effects or additive effects of rTMS were observed. The present study demonstrates that VR-based no-go training can effectively regulate food-related responses and extends earlier work by demonstrating robust inhibition training effects across implicit and explicit measures, while highlighting the importance of considering individual differences in future research.

## 1 Introduction

The global prevalence of overweight and obesity has increased rapidly, more than doubling since 1990 (1), which poses serious public health concerns. Importantly, maladaptive eating is not limited to individuals with elevated body weight; many people often struggle to control their impulsive cravings for high-calorie foods rich in fats and sugars, partly due to the intrinsically rewarding nature of these foods (2). Various strategies have been suggested to help control eating behavior (3, 4). Among them, food inhibitory control training (ICT) has received increasing research attention (5, 6).

ICT training involves repetitive association of food cues with inhibition signals, which may help diminish automatic responses to food and cultivate an implicit goal of inhibiting the desire to consume food (7, 8). The training procedures are typically implemented using a modified version of the go/no-go task. In the task, participants are instructed to press a response button for an image paired with a go cue but hold the response for an image paired with a no-go cue. During food-inhibition training, unhealthy high-calorie food images are consistently paired with no-go cues, thereby reinforcing the association between such foods and inhibitory control. Recent meta-analyses of studies examining the effects of inhibition training on food consumption and food choice behavior reported modest effect sizes, suggesting scope for further improvement of training approaches (5, 6).

With the aim of improving intervention outcomes, attention has been increasingly directed toward combining multiple approaches (9, 10). One such approach involves the use of repetitive transcranial magnetic stimulation (rTMS) to modulate brain regions involved in target functions. The dorsolateral prefrontal cortex (DLPFC) has received increasing attention as a stimulation target in studies of dietary control due to its implication in top-down regulatory processes, including inhibitory control (11–13). Particularly, meta-analytic findings indicate that the left DLPFC has been more frequently targeted in rTMS studies (14) and is associated with greater effect sizes in regulating food craving and consumption compared to right-sided stimulation (15). In our previous study, we combined no-go inhibition training with high-frequency rTMS to enhance intervention effects. We found that healthy women who received both high-frequency rTMS and no-go inhibition training exhibited reduced chocolate intake during a bogus taste test, compared to those who received either no-go or rTMS alone (9). However, the generalizability of these findings is limited due to the small sample size (i.e., n = 15 per group), and meta-analytic evidence across a range of cognitive domains suggests that the effects of combined intervention are inconsistent (16).

Building on our previous work (9), the current study employs a larger sample size and incorporates several methodological extensions aimed at improving the effectiveness of the intervention. First, the no-go inhibition training was implemented in a virtual reality (VR) environment. As no-go training relies on simple associations between stimulus and response inhibition, participants may become easily distracted and bored during the conventional training tasks (17–19). Accordingly, there is a growing recognition of the importance of enhancing engagement and adherence in cognitive interventions (20). VR environment can provide more interactive and ecologically-valid experience during training in this regard (21). Specifically, compared to conventional inhibition training, VR can better simulate real-life exposure to tempting foods, enable inhibition of real motor actions (e.g., reaching and grasping), and increase the sense of presence (22). These features can create more engaging and challenging training experience, which may in turn facilitate more effective training. Consistently, earlier studies applying VR techniques for cognitive training have reported improved task performance and clinical efficacy across a range of clinical conditions, including eating disorders as well as other psychiatric and neurological conditions (22–25).

Second, we included implicit attitudes toward food items as one of the primary outcome measures. Implicit attitudes toward food have been identified as reliable predictors of food choices and eating behavior (26–29). As revealed by recent reviews, the majority of previous studies on association-based cognitive training have reported changes in implicit attitudes favoring healthier foods, though some inconsistencies remain (8, 30, 31). According to the Behavior Stimulus Interaction (BSI) theory, no-go training induces a conflict between the automatic response tendency to tempting foods and the requirement to inhibit responding, which is resolved through food cue devaluation (32). Such devaluation may be reflected in changes in implicit evaluative attitudes toward food cues, which may in turn contribute to subsequent reductions in craving and intake of tempting foods (8, 33).

In addition, the current study employed personalized training using food items tailored to individual preferences identified during screening, together with a pre–post design to assess changes following the intervention. These approaches were intended to reduce individual variability that might obscure intervention effects and to enhance the reliability of the results (34). To further minimize sex-related confounding influences, the current study included only female participants. Prior evidence suggests that women and men differ in the experience of food craving and its regulation. Specifically, women report more frequent and stronger food craving than men, particularly for sweet, palatable foods, and exhibit greater difficulty in regulating these cravings (35). Furthermore, the study recruited healthy individuals with elevated preference for high-calorie foods, allowing us to examine whether the intervention modulates the heightened craving and choice for such foods. This approach also ensured methodological consistency with our previous study (9).

In the current study healthy young women preferring high-calorie foods were randomly assigned to one of four groups in a 2 (rTMS: active vs. sham) × 2 (training: no-go vs. neutral) between-subjects design. High-frequency rTMS was applied to the left DLPFC region. VR-based no-go training was delivered using individually preferred high-calorie food items as no-go target stimuli. Participants’ craving and implicit attitudes toward high- and low-calorie food items were assessed before and after the intervention, and a food choice task was administered at the end. We hypothesized that no-go training would reduce craving and implicit preference for high-calorie foods and facilitate healthy food choices, and that these effects would be enhanced when combined with active rTMS. In addition, given that baseline preference may influence food craving and consumption, and that the luteal phase of the menstrual cycle is associated with increases in food craving and consumption in women (35), we conducted exploratory analyses to examine whether these factors moderated the intervention effects.

## 2 Materials and methods

### 2.1 Participants

A total of 121 right-handed healthy adult women (age range, 18~39 years) participated in the current study. This decision to include only female participants was particularly relevant in the present study, which employed individually tailored inhibition targets. Participants were recruited through online advertisement to the local community. The inclusion criteria were as follows. First, participants had a score of 64 or higher on the General-Food Craving Questionnaire-Trait (G-FCQ-T), corresponding to one standard deviation above the mean in the Korean validation study (36, 37). Second, participants reported a preference score of 7 or higher on a 9-point Likert scale for at least one of the following foods: bread, chocolate, and savory snacks in a General Preference Rating Scale. Details of the scale are provided in **Supplementary Table 1**. Based on TMS safety guidelines (38), participants with a current or past diagnosis of psychiatric or neurological disorders, pregnancy, or the presence of implanted metal devices (e.g., pacemakers and metal braces) were excluded. All participants provided written informed consent before participation. The study protocol was approved by the Korea University Institutional Review Board (KUIRB-2023-0063), and all participants received monetary compensation for their time and effort.

Participants were randomly assigned to one of four groups: active rTMS combined with no-go training (active/no-go), active rTMS combined with neutral training (active/neutral), sham stimulation combined with no-go training (sham/no-go), and sham stimulation combined with neutral training (sham/neutral). The sample size was determined using G*Power version 3.1.9.7 (39) based on a previous study (9) showing an effect size of f = 0.27 for the interaction effect between rTMS and inhibition training. Together with power of 0.80 and α of 0.05, the yielded sample size was 107. A total of 121 participants were recruited to account for potential data loss or drop-out. One participants withdrew during the rTMS phase because of discomfort. Although dietary status was not an exclusion criterion, two participants were excluded because self-reported dietary restrictions directly involved the high-calorie target foods used in the go/no-go training (e.g., bread/carbohydrates or limiting salty and unhealthy foods), and thus they were not the intended target population. As a result, the final sample of 118 was included in the data analysis. The distribution of the final sample across target food categories is presented in **Supplementary Table 2**.

### 2.2 High-frequency repetitive transcranial magnetic stimulation

A single session of rTMS was administered using a Magstim Rapid2 (Magstim, Whitland, UK) with a double 70mm figure-of-eight air film coil. The stimulation procedures and parameters were identical to those used in our early study demonstrating stimulation effects on food intake (9). Specifically, the stimulation was applied to the left DLPFC at a frequency of 10 Hz and an intensity of 40% of the maximal stimulator output. A fixed stimulation intensity was used instead of motor threshold-based intensity, based on evidence that motor threshold does not reliably reflect the excitability of non-motor cortical regions (40, 41). In addition, a recent meta-analytic study indicated that fixed intensity methods have been used in a substantial proportion of studies (i.e., 27%) and result in effects comparable to those with resting motor threshold methods (42). The stimulation site was defined as F3 of the international 10–20 system (43). Four blocks of stimulation were applied, with a one-minute break between each block. Each block consisted of 10 trains, with each train lasting 3 seconds and a 7-second inter-train interval. In total, 1,200 pulses were delivered over 10 minutes. During the sham stimulation, the coil was positioned perpendicular to the scalp.

### 2.3 Virtual reality-based no-go training

**Figure 1:**
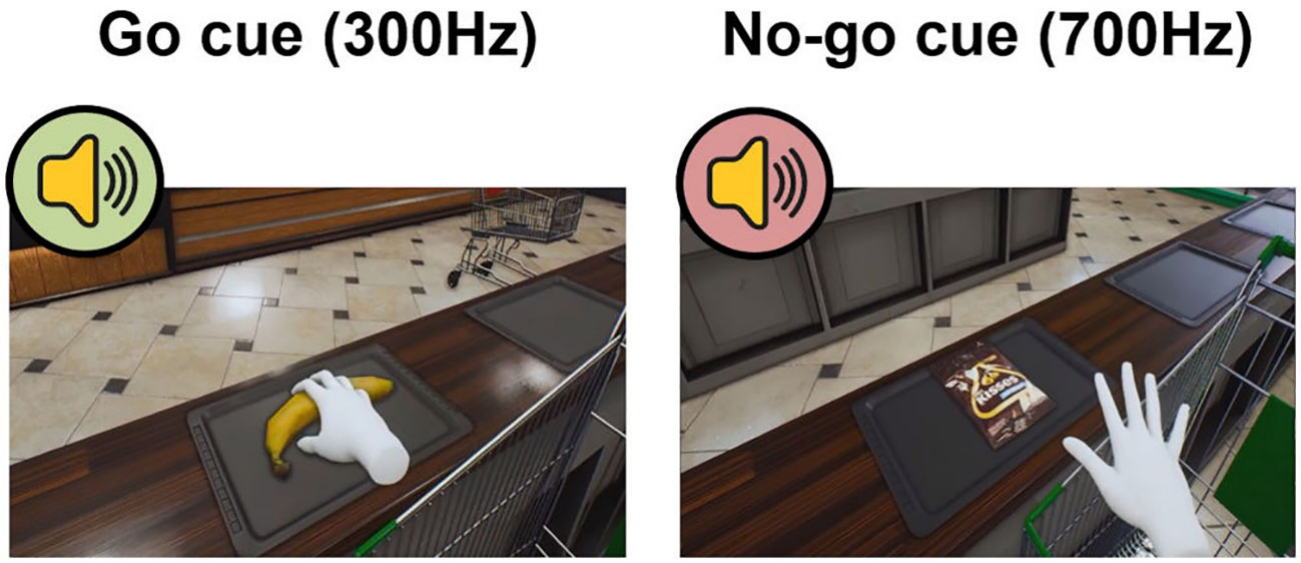
Example go and no-go screenshots from the VR-based no-go training task.

The VR-based no-go training was implemented in a virtual grocery store environment designed to simulate real-life food choice situations (**Figure 1**). The VR task was created using Unreal Engine 4 (Epic Games, Cary, NC, USA) and performed by connecting with the Oculus Quest 2 head-mounted display (HMD; Meta Platforms, Menlo Park, CA, USA). The task involved the presentation of food items or everyday objects that participants had to either pick up or pass by, depending on distinct auditory cues. Three stimulus types were used: high-calorie foods, low-calorie foods, and everyday objects, with each type containing eight distinct items (**Supplementary Figure 1**). High-calorie foods included items from bread, chocolate, or snack categories, low-calorie foods consisted of fruits and vegetables, objects were common everyday items including a camera and a book. Low-calorie foods and objects were identical for all participants, whereas high-calorie foods were tailored individually based on each participant’s food preference ratings obtained during recruitment. That is, if a participant reported the highest preference for bread, all high-calorie items were selected from the bread category. The same approach was applied for those who preferred chocolate or snacks.

During the task, items were presented one by one on a grocery store counter. Each item was accompanied by a simple sound tone of different frequency, serving as a go (i.e., 300 Hz) or no-go cue (i.e., 700 Hz). For a go cue, participants were instructed to grab the item and put it in their own cart, whereas for a no-go cue, they were instructed not to grab the item and pass it. The no-go and neutral groups differed in food-cue assignments. In the no-go group, high-calorie foods were always paired with a no-go cue, and low-calorie foods were always paired with a go cue. In contrast, in the neutral group, high- and low-calorie food items were paired with a go or no-go cue at equal frequencies. Objects were likewise paired with a go or a no-go cue equally often. For each category, the eight unique items were repeated six times, resulting in a total of 144 trials. The task lasted approximately 10 minutes. Participants completed 20 practice trials before proceeding to the training task.

### 2.4 Single category implicit association tests

Two SCIATs were prepared to measure implicit attitudes toward two distinct food categories: high-calorie foods and low-calorie foods. Unlike the traditional Implicit Association Test (IAT), which assesses relative implicit attitudes between two target categories, the SCIAT measures the implicit attitude toward a single target category (44). The target category for the high-calorie food SCIAT was chocolate, bread, or snacks, depending on the participant’s preference, while the low-calorie food SCIAT used fruits/vegetables as the target category. The attribute categories represented affective valence and consisted of positive and negative words. Target category stimuli were presented as images, whereas attribute category stimuli were words. The target category consisted of six images selected from the eight items used in the VR-based no-go training. The primary difference from the VR-based no-go training stimuli is that the VR items were presented as 3D objects, whereas the SCIAT images were 2D. Six positive (joy, satisfaction, refreshment, liking, pleasure, fantastic) and six negative (discomfort, dissatisfaction, disappointment, regret, rejection, indifference) attribute words were used (**Supplementary Table 3**). All attribute words were selected from the standardized Korean affect word list (45).

Each SCIAT consisted of four blocks. The first and third blocks were practice blocks, each consisting of 24 trials. Participants practiced classifying attribute category words and target category images using the left (‘z’) and right (‘/’) response keys. Category labels were presented at the top left and top right of the screen, and each stimulus appeared at the center on each trial. The second and fourth blocks served as test blocks, each consisting of 72 trials. Participants were instructed to respond as accurately and quickly as possible. For incorrect responses, a red ‘X’ appeared at the bottom of the screen, and participants were required to press the correct key before proceeding to the next trial. In all blocks, the proportion of stimuli was deliberately unequal: target category images, attribute words requiring the same response as the target images, and attribute words requiring the opposite response were presented in a fixed 7:7:10 ratio. This imbalance was introduced to reduce the differences in the number of left and right responses. The response assignment of the food target to either the positive (i.e., compatible) or negative (i.e., incompatible) attribute category was counterbalanced across participants so that half of the participants first performed the compatible assignment (i.e., ‘food’, ‘positive’ versus ‘negative’) before the incompatible assignment (i.e., ‘positive’ versus ‘food’, ‘negative’). The other half participants performed the task in the reverse order. The high-calorie food SCIAT was always administered first followed by the low-calorie food SCIAT. The task was programmed and administered using E-Prime 3.0 (Psychology Software Tools, Pittsburgh, PA, USA).

The SCIAT score was calculated using the D2 suggested by Greenwald et al. (46), with adaptations for the SCIAT (44). Participants with more than 10% of their responses faster than 300 ms were excluded from the SCIAT analysis. In our sample, one participant exceeded this threshold in the low-calorie food SCIAT. Therefore, analyses for the low-calorie food SCIAT included 117 participants. Analysis for the high-calorie food SCIAT included all 118 participants. For scoring, trials with response time below 350 ms or above 1500 ms were removed. Error-trial RT was replaced with the latency of the correct response made after the error. Scores were computed separately for the practice and test blocks. For each block type, the score was calculated by subtracting the mean RT of the compatible block (‘food’ + ‘positive’ versus ‘negative’) from the mean RT of the incompatible block (‘positive’ versus ‘food’ + ‘negative’), and then dividing this difference by the pooled standard deviation of the two blocks. The final SCIAT score was obtained by averaging the two values (practice block score and test block score). Greater positive scores indicate faster responses in the compatible block relative to the incompatible block, reflecting a stronger implicit association between food and positive attributes.

Consistent with prior studies using SCIATs (47–49), statistical analyses were conducted separately for high-calorie and low-calorie food SCIAT tasks.

### 2.5 Craving rating task

The craving rating task was conducted to measure explicit craving rating for high- and low-calorie foods. Stimuli were three types of images: high-calorie foods, low-calorie foods, and everyday objects. Each category included 16 distinct images, half of which were used in the VR-based no-go training, and the other half were not used in the training to assess whether the training effects generalized beyond the trained items. The task consists of 48 trials. Each trial of the task started with a blank screen displayed for 1500 ms, followed by a fixation cross for 1000ms. Then, an image was presented on a black background, accompanied by the question (“How much do you want to eat/use this item now?”). Participants responded on a visual analog scale of –100 (“Not at all”) to 100 (“Very much”). Then they pressed the “Next” button to proceed to the next trial. The task was programmed and administered using E-Prime 3.0 (Psychology Software Tools, Pittsburgh, PA, USA).

### 2.6 Food choice task

To assess food choice in a context resembling real-world decision-making, participants were presented with high- and low-calorie food items simultaneously, a measure recognized as a strong predictor of actual food intake. The stimuli consisted of four types: trained high-calorie, trained low-calorie, untrained high-calorie, and untrained low-calorie items. The trained items were identical to those used during the training. Each type included two distinct items, resulting in a total of eight food items presented together in a single display. Participants viewed images of the eight food items simultaneously on a computer screen and were instructed to select up to two items they would like to consume and to take home. They were informed that a small package containing their selected items would be provided before leaving the laboratory. These instructions were intended to encourage participants to make choices based on their consumption preferences, thereby enhancing the internal validity of the task.

Choice scores were calculated by assigning ‘–1’ point for choosing a high-calorie food and ‘+1’ point for choosing a low-calorie food. If no food was chosen, the score was ‘0’. The total food choice scores were calculated as the sum of the two choices, with higher score indicating preference for low-calorie foods over high-calorie foods.

### 2.7 Self-reported questionnaires

The Positive Affect and Negative Affect Scale (PANAS) was used to assess the current positive and negative affective states (50). Depressive symptoms were assessed using the Beck Depression Inventory (BDI (51);, and anxiety levels were measured with the State–Trait Anxiety Inventory (STAI (52);. Current levels of perceived stress were evaluated using the Perceived Stress Scale (PSS (53);. Sensitivity to punishment and reward was assessed using the Behavioral Inhibition System/Behavioral Approach System scales (BIS/BAS (54);.

Eating disorder–related characteristics were measured using the Eating Disorder Inventory–2 (EDI–2), a 23-item measure assessing a broad range of cognitive, emotional, and behavioral features associated with disordered eating (55, 56). Dietary restraint was assessed using the Restraint Scale (RS), a 10-item measure reflecting the cognitive efforts to resist eating impulses and the tendency to eat less than desired (56, 57).

### 2.8 Procedures

Participants were instructed to fast for 3 hours before the start of the study to control for hunger, which has been shown to influence neural and behavioral responses to food cues (58, 59). Upon arrival, they provided written informed consent and reported the time since their last meal. They first completed the high-calorie food SCIAT, followed by the low-calorie food SCIAT, with a 1-minute break in between. Next, participants performed the craving rating task and completed a set of questionnaires assessing current affective states, stress, reward and punishment sensitivity, eating-related characteristics, and dietary restraint. Following these assessments, participants received either high-frequency rTMS or sham stimulation for approximately 10 minutes, depending on group assignment. After a 5-minute break, participants performed the VR-based no-go training task. Participants then completed the Presence Questionnaire (PQ (60); to assess perceived presence in the virtual environment, followed by the PANAS. They subsequently repeated the high-calorie food SCIAT, the low-calorie food SCIAT, and craving rating task in the same order as at baseline. Finally, the food choice task was administered. Before debriefing, participants reported their menstrual cycle information and provided their height and weight, which were used to calculate body mass index (BMI).

### 2.9 Statistical analyses

All statistical tests were conducted using SPSS version 29 (IBM Corp., Armonk, NY, USA). Outcome measures were primarily analyzed using factorial ANOVAs, with stimulation (active vs. sham) and training (no-go vs. neutral) as between-subject factors and time (pre vs. post) as a within-subject factor. Additional task-specific factors were considered as appropriate. For the food choice task, analyses included only between-subject factors.

Additional exploratory analyses were conducted to further examine potential moderators. To examine whether baseline target food preference or menstrual cycle phase moderated the intervention effects, moderation analyses were conducted using PROCESS macro in SPSS (Model 1, version 4.2 (61);. The dependent variables were three outcome measures that showed training effects in the primary analyses: high-calorie food SCIAT scores, low-calorie food craving scores, and food choice scores. For the SCIAT and craving measures, pre-post change scores (post-intervention minus pre-intervention) were calculated and entered as dependent variables in the analyses. The training condition was entered as the independent variable, with baseline target food preference or menstrual cycle phase (coded as luteal vs. non-luteal) as moderators. Participants who reported being unsure of their menstrual cycle phase (n=8) were excluded from moderation analyses involving menstrual cycle. Interaction terms between training condition and moderator were also included in the model.

## 3 Results

### 3.1 Participants characteristics

**Table 1** shows the means and standard deviations of the descriptive variables for each group, along with the group test statistics. One-way ANOVAs for each descriptive variable revealed that groups were similar for various measures: age, BMI, general food cravings trait, high-calorie food preference scores, fasting time, and questionnaire scores including BDI, PSS, STAI-State, STAI-Trait, BIS, BAS-Reward responsiveness, BAS-Drive, BAS-Fun seeking, EDI2-Drive for thinness, EDI2-Body dissatisfaction, EDI2-Bulimia, RS and PQ, all *F*s < 1.30, all *p*s > 0.25 (see **Table 1** for the complete list).

**Table 1:** Means and standard deviations of demographic characteristics and self-reported questionnaire scores by group, along with one-way ANOVA test statistics.

Measures	Active/no-go	Active/neutral	Sham/no-go	Sham/neutral	Test statistics	
	*M* ± *SD*	*M* ± *SD*	*M* ± *SD*	*M* ± *SD*	*F* (3,114)	*p*
Participant characteristics						
Age	25.03 ± 4.63	24.70 ± 5.14	24.80 ± 5.38	24.10 ± 4.43	0.19	.904
BMI	22.32 ± 4.35	22.17 ± 4.19	21.17 ± 2.26	22.53 ± 3.36	0.83	.483
G-FCQ-T	81.07 ± 12.93	81.27 ± 12.49	82.90 ± 13.52	81.83 ± 14.27	0.11	.952
High-calorie food preference	8.17 ± 0.80	8.10 ± 0.76	8.03 ± 0.85	8.17 ± 0.76	0.21	.890
Fasting time	8.12 ± 5.54	7.49 ± 4.78	6.63 ± 5.10	7.78 ± 5.87	0.43	.735
BDI	9.10 ± 5.55	8.93 ± 6.59	11.17 ± 6.56	10.00 ± 7.50	0.72	.542
PSS	23.00 ± 5.61	21.87 ± 6.53	24.40 ± 5.71	23.10 ± 6.84	0.84	.475
STAI						
State	41.79 ± 9.66	40.53 ± 8.62	44.83 ± 8.65	43.90 ± 10.92	1.27	.290
Trait	46.90 ± 10.22	46.57 ± 10.41	49.67 ± 10.28	46.34 ± 11.31	0.64	.590
BIS/BAS						
BIS	19.79 ± 3.40	19.67 ± 4.59	21.47 ± 4.19	20.34 ± 4.73	1.11	.349
BAS-Reward responsiveness	15.62 ± 2.47	16.07 ± 2.35	15.70 ± 2.96	16.38 ± 2.60	0.53	.664
BAS-Drive	11.24 ± 2.69	11.90 ± 2.59	11.30 ± 2.38	11.48 ± 2.49	0.41	.747
BAS-Fun seeking	10.93 ± 2.63	11.13 ± 2.74	10.93 ± 2.72	10.79 ± 2.80	0.08	.972
EDI-2						
Drive for thinness	7.83 ± 5.79	8.43 ± 5.46	9.17 ± 5.84	9.45 ± 6.10	0.47	.707
Body dissatisfaction	9.90 ± 6.12	9.00 ± 6.12	10.20 ± 5.79	11.21 ± 7.09	0.62	.606
Bulimia	5.59 ± 4.67	4.53 ± 4.17	5.67 ± 4.75	5.21 ± 4.36	0.39	.757
Restraint Scale	16.38 ± 5.96	16.87 ± 5.45	17.90 ± 5.38	17.66 ± 5.23	0.48	.698
PQ	93.28 ± 9.52	90.17 ± 10.29	90.93 ± 10.11	92.10 ± 9.69	0.56	.646

G-FCQ-T , General-Food Craving Questionnaire-Trait; BDI , Beck Depression Inventory; PSS , Perceived Stress Scale; STAI , State–Trait Anxiety Inventory; BIS, Behavioral Inhibition System; BAS , Behavioral Approach System; EDI–2 , Eating Disorder Inventory–2; PQ , Presence Questionnaire.

### 3.2 Intervention-dependent effects in outcome measures

**Table 2** presents the means and standard deviations of all outcome measures for each group.

**Table 2:** Means and standard deviations of all outcome measures by group.

Measures		Active/no-go	Active/neutral	Sham/no-go	Sham/neutral
Mood					
Positive affect	Pre	26.14 ± 6.05	28.80 ± 7.85	27.60 ± 6.39	29.31 ± 8.37
	Post	31.17 ± 6.87	31.70 ± 7.81	33.40 ± 7.81	31.28 ± 8.82
Negative affect	Pre	15.83 ± 5.25	17.17 ± 6.75	16.37 ± 5.06	17.00 ± 5.86
	Post	11.41 ± 2.47	12.73 ± 4.34	11.83 ± 2.47	12.83 ± 3.21
Implicit attitudes					
High-calorie	Pre	0.13 ± 0.71	0.04 ± 0.43	0.28 ± 0.41	–0.09 ± 1.04
	Post	0.05 ± 0.39	0.13 ± 0.45	0.04 ± 0.50	0.10 ± 0.43
Low-calorie	Pre	0.10 ± 0.41	0.04 ± 0.32	–0.03 ± 0.73	0.06 ± 0.36
	Post	0.14 ± 0.39	0.11 ± 0.46	0.13 ± 0.35	0.07 ± 0.37
Craving					
High-calorie	Pre	39.88 ± 39.06	39.05 ± 31.49	32.26 ± 33.80	39.53 ± 35.04
	Post	36.44 ±. 40.94	40.72 ± 33.80	34.22 ± 39.79	35.51 ± 38.81
Low-calorie	Pre	15.95 ± 29.69	19.61 ± 28.78	9.04 ± 28.06	12.07 ± 30.28
	Post	19.03 ± 32.57	17.34 ± 32.54	14.04 ± 29.20	7.52 ± 31.27
Choice		–0.21 ± 1.35	–0.87 ± 1.11	–0.23 ± 1.38	–0.52 ± 1.21

#### 3.2.1 SCIAT for high-calorie and low-calorie foods

**Figure 2:**
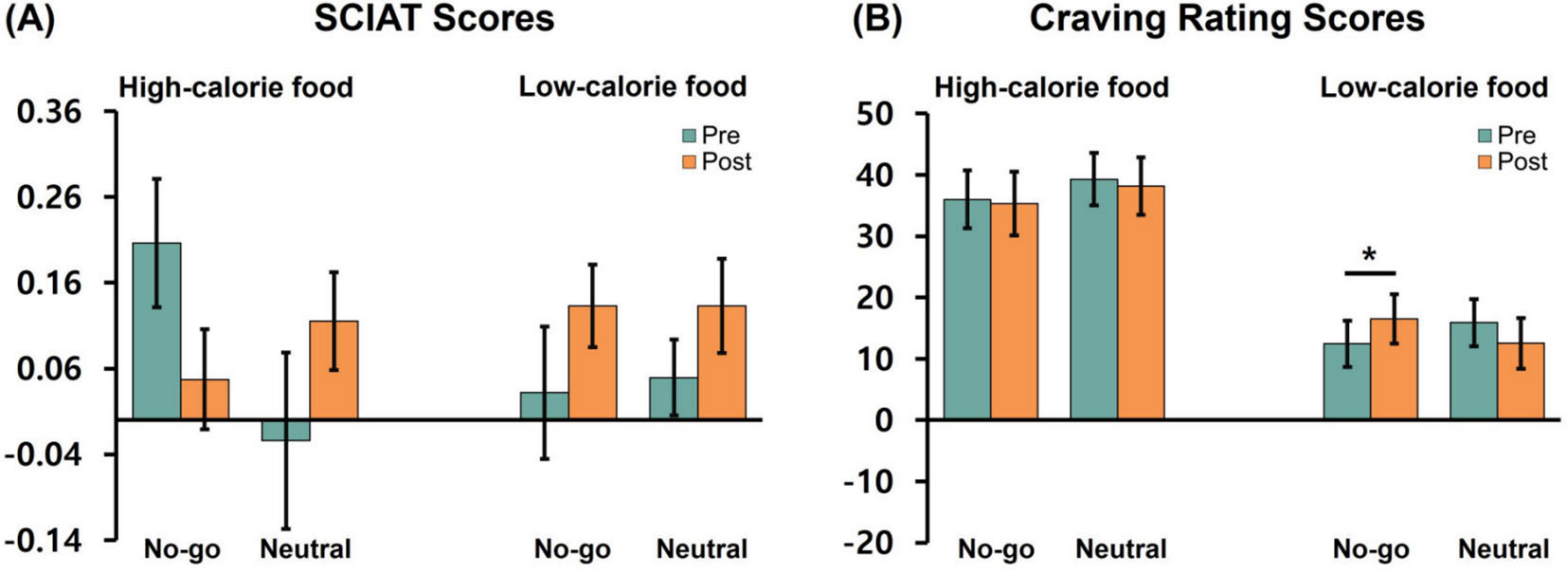
SCIAT scores **(A)** and food craving ratings **(B)** as a function of food type, training condition, and time (pre vs. post). Error bars represent standard errors of the mean. * indicates p <.05.

For the high-calorie food SCIAT, a 2 (stimulation: active vs. sham) × 2 (training: no-go vs. neutral) × 2 (time: pre vs. post) ANOVA revealed a significant interaction between training and time, *F (*1,114*)* = 4.61, *p* = .034, $\eta_{p}^{2}$ = 0.04. To reveal the nature of this interaction, follow-up comparisons were conducted separately for each training group. In the no-go training group, the IAT score showed a marginal decrease from pre (*M* = 0.21, *SD* = 0.57) to post (*M* = 0.05, *SD* = 0.45), *t (*58*)* = 1.84, *p* = .072 (**Figure 2A**). In the neutral training group, there was no significant change between pre (*M* = -0.02, *SD* = 0.79) and post (*M* = 0.12, *SD* = 0.44), *t*(58) = –1.30, *p* = .199. Other main or interaction effects were not significant, all *F*s < 1.0, all *p*s > 0.3.

For the low-calorie food SCIAT, a 2 (stimulation: active vs. sham) × 2 (training: no-go vs. neutral) × 2 (time: pre vs. post) ANOVA revealed no significant main or interaction effects, all *F*s < 2.3, all *p*s > 0.1.

#### 3.2.2 Craving rating task

For the craving rating task, a 2 (stimulation: active vs. sham) × 2 (training: no-go vs. neutral) × 2 (food type: high-calorie food vs. low-calorie food) × 2 (time: pre vs. post) ANOVA was conducted. There was a significant main effect of food type, *F*(1,114) = 40.85, *p* <.001, $\eta_{p}^{2}$ = 0.26, with higher craving ratings for high-calorie foods (*M* = 37.19, *SD* = 35.04) than for low-calorie foods (*M* = 14.34, *SD* = 2.70). No other main or interaction effects were significant, all *F*s < 2.4, all *p*s > 0.1, except for a marginally significant training × food type × time interaction, *F*(1,114) = 3.67, *p* = .058, $\eta_{p}^{2}$ = 0.03.

To explore this three-way interaction, follow-up analyses were conducted separately for each food type using 2 (training) x 2 (time) ANOVAs. For high-calorie foods, no significant main or interaction effects were observed, all *F*s < 0.3, all *p*s > 0.6. For low-calorie foods, there was a significant training × time interaction, *F*(1,116) = 7.80, *p* = .006, $\eta_{p}^{2}$ = 0.06. Follow-up analyses revealed that, in the no-go training group, craving ratings significantly increased from pre (*M* = 12.44, *SD* = 28.83) to post (*M* = 16.49, *SD* = 30.74), *p* = .034 (**Figure 2B**). In contrast, the neutral training group showed a marginal decrease from pre (*M* = 15.90, *SD* = 29.52) to post (*M* = 12.51, *SD* = 32.03), *p* = .075. The main effects of training and time were not significant, all *F*s *<*0.1, all *p*s > 0.8.

#### 3.2.3 Food choice task

**Figure 3:**
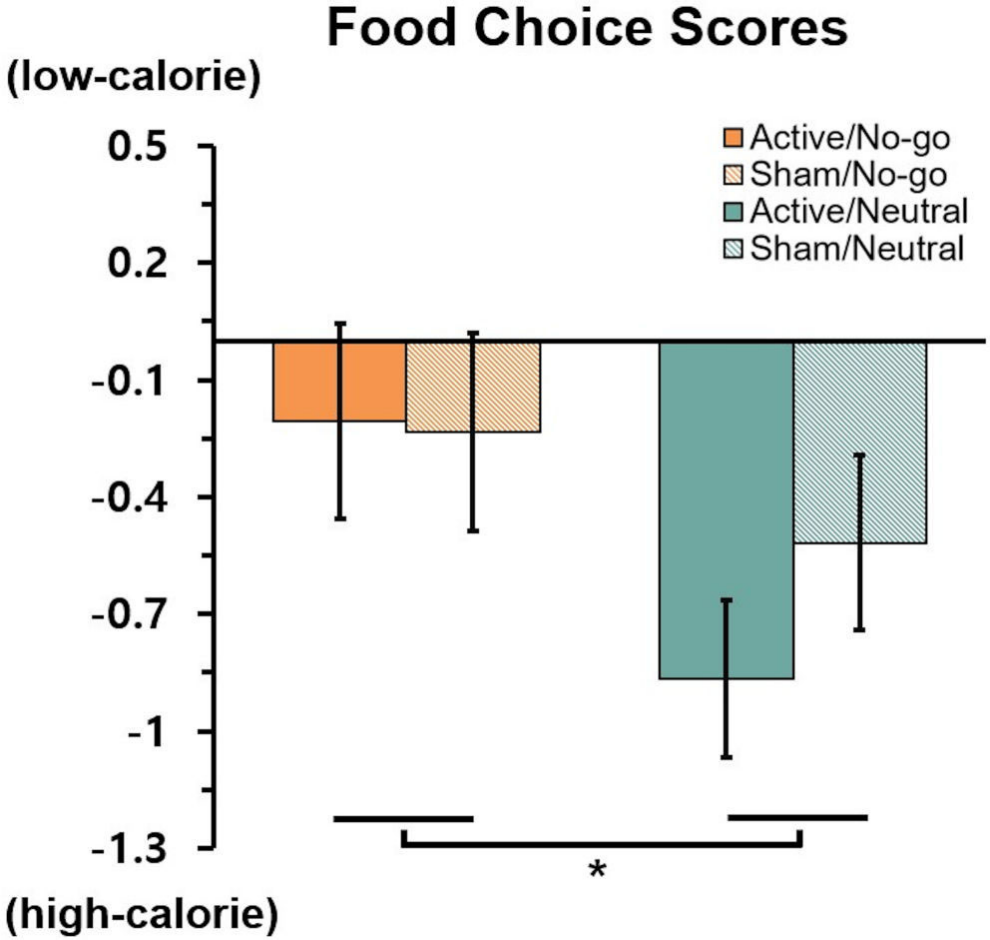
Food choice scores by group. * indicates p < 05.

Food choice scores entered a 2 (stimulation: active vs. sham) × 2 (training: no-go vs. neutral) ANOVA. The negative values indicate choosing more high-calorie foods over low-calorie foods, whereas positive values reflect the opposite pattern. There was a significant main effect of training, *F*(1,114) = 4.09, *p* = .045, $\eta_{p}^{2}$ = 0.04, indicating that the no-go training group showed higher food choice scores (*M* = –0.22, *SD* = 1.35) compared to the neutral training group (*M* = –0.69, *SD* = 1.16). The no-go training group chose less high-calorie foods than the neutral group. No other main effect or interaction effect, all *F*s < 0.7, *p*s > 0.4 (**Figure 3**).

#### 3.2.4 Positive and negative affect scores

For affect scores, a 2 (stimulation: active vs. sham) × 2 (training: no-go vs. neutral) × 2 (affect: positive vs. negative) × 2 (time: pre vs. post) ANOVA was conducted. There was a significant main effect of affect, *F*(1,114) = 452.03, *p* <.001, $\eta_{p}^{2}$ = 0.80, with higher scores for positive affect (*M* = 29.93, *SD* = 6.91) than for negative affect (*M* = 14.40, *SD* = 4.17). There were significant interactions of training × time, *F*(1,114) = 6.05, *p* = .015, $\eta_{p}^{2}$ = 0.05; affect × time, *F*(1,114) = 127.25, *p* <.001, $\eta_{p}^{2}$ = 0.53; training × affect × time, *F*(1,114) = 4.58, *p* = .034, $\eta_{p}^{2}$ = 0.04.

**Figure 4:**
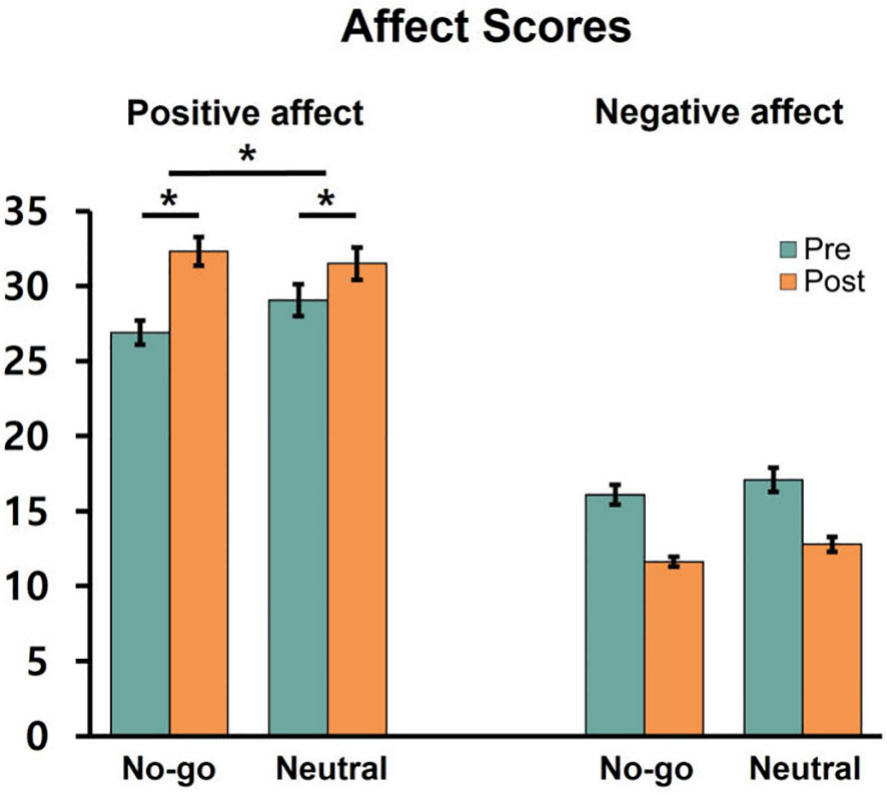
Positive and negative affect ratings as a function of food type, training condition, and time (pre vs. post). Error bars represent standard errors of the mean. * indicates p <. 01.

To further examine the significant three-way interaction, follow-up analyses were conducted separately for each affect using 2 (training) x 2 (time) ANOVAs. For positive affect, there was a significant main effect of time, *F*(1,116) = 54.23, *p* <.001, $\eta_{p}^{2}$ = 0.32, indicating an increase from pre (*M* = 27.97, *SD* = 7.24) to post (*M* = 31.90, *SD* = 7.81). The main effect of training was not significant, *F*(1,116) = 0.28, *p* = .596. The training × time interaction was also significant, *F*(1,116) = 7.80, *p* = .006, $\eta_{p}^{2}$ = 0.06 (**Figure 4**). Follow-up analyses revealed that both the no-go and neutral training groups showed significant increases from pre (no-go: *M* = 26.88, *SD* = 6.21; neutral *M* = 29.05, *SD* = 8.04) to post (no-go: *M* = 32.31, *SD* = 7.38; neutral: *M* = 31.49, *SD* = 8.26), *p*s <.002. A direct comparison of the post-pre change scores between groups revealed a significant difference, *t*(116) = 2.79, *p* = .006, with a greater increase in the no-go than in the neutral training group.

For negative affect, there was a significant main effect of time, *F*(1,116) = 133.68, *p* <.001, $\eta_{p}^{2}$ = 0.54, indicating a decrease from pre (*M* = 16.59, *SD* = 5.72) to post (*M* = 12.20, *SD* = 3.23). No other effects were significant, all *F*s < 0.1, all *p*s > 0.8.

### 3.3 Individual characteristics as moderators

#### 3.3.1 Baseline target food preference

**Figure 5:**
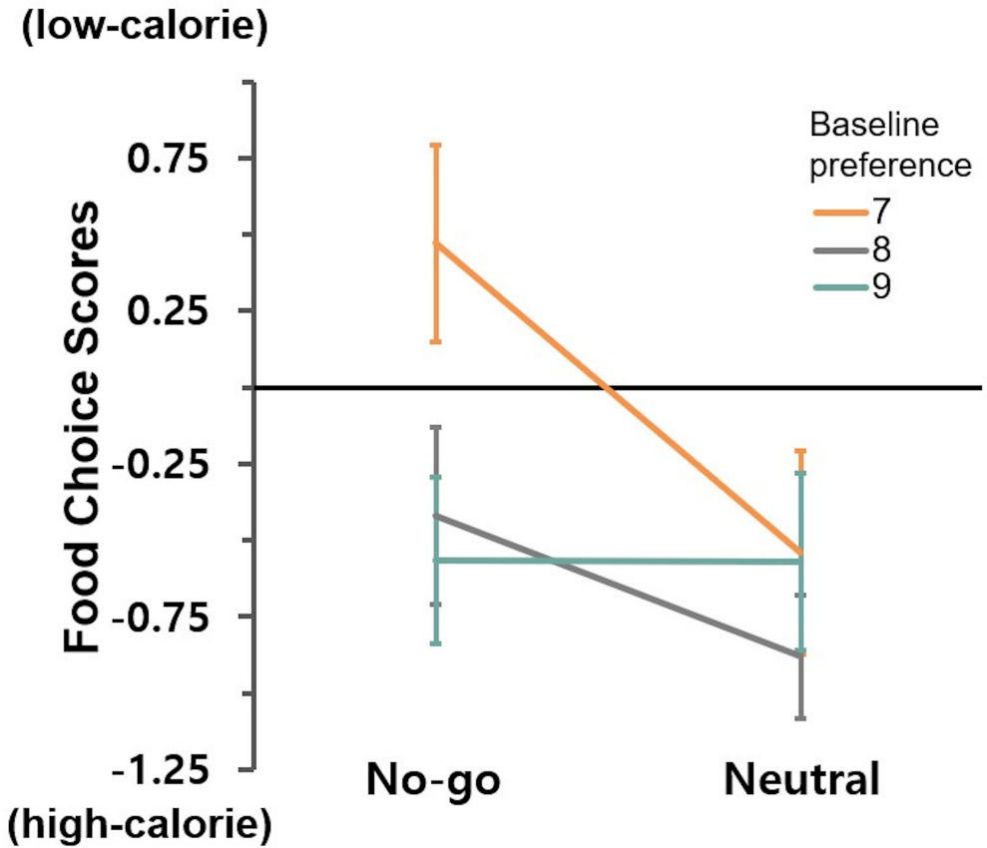
Moderating effect of baseline preference for trained foods on food choice.

We did not observe a significant moderating effect of target food preference on the relationship between training condition and changes in implicit attitudes toward high-calorie foods, *B* = –0.03, *t*(114) = –0.39, *p* = .700. Similarly, no moderation effect was found for changes in low-calorie food craving, *B* = –0.62, *t*(114) = –0.36, *p* = .719. For choice scores, we found a marginally significant interaction effect, *B* = –0.26, *t*(114) = –1.77, *p* = .080, suggesting a trend-level moderation effect of preference level (**Figure 5**). A simple slopes analysis revealed that intervention effect was significant for participants with preference level of 7, *B* = 0.52, *SE* = 0.20, 95% CI = [0.13, 0.92], *t*(114) = 2.62, *p* = .010, and 8, *B* = 0.26, *SE* = 0.12, 95% CI = [0.04, 0.49], *t*(114) = 2.29, *p* = .024. No intervention effect was found for individuals with preference level of 9, *B* = 0.01, *SE* = 0.17, 95% CI = [–0.34, 0.35], *t*(114) = 0.03, *p* = .974.

#### 3.3.2 Menstrual cycle

The menstrual cycle did not moderate the relationship between training condition and changes in implicit attitudes toward high-calorie foods, *B* = –0.05, *t*(106) = –0.64, *p* = .522. Similarly, no moderation effect was found for changes in low-calorie food craving, *B* = 1.12, *t*(106) = 0.82, *p* = .417. In contrast, a significant moderation effect of the menstrual cycle was observed for food choice, *B* = 0.32, *t*(106) = 2.71, *p* = .008 (**Supplementary Figure 2**). A simple slopes analysis showed that for participants in the luteal phase, the effect of the training condition on food choice was significant, *B* = 0.52, *SE* = 0.18, 95% CI = [0.17, 0.87], *t*(106) = 2.94, *p* = .004, whereas for participants in the non-luteal phase, the effect was not significant, *B* = –0.11, *SE* = 0.15, 95% CI = [–0.42, 0.19], *t*(106) = –0.74, *p* = .458.

## 4 Discussion

In the current study, we investigated whether cognitive inhibition training combined with brain stimulation modulates implicit food-related attitudes, craving ratings and food-choice behaviors. In particular, we aimed to determine whether the combined intervention results in additive effects beyond those of each intervention administered independently. We found consistent effects of no-go inhibition training across outcome measures. Participants who received no-go training showed reduced positive implicit attitudes toward high-calorie foods and increased craving for low-calorie foods following training, and made healthier food choices than those in the neutral training. In addition, the effects of no-go training on healthier food choices were moderated by preexisting preference for high-calorie foods and menstrual cycle phase. Furthermore, no-go training resulted in a greater pre-to-post increase in positive affect compared to neutral training. However, we observed no significant effects of rTMS and no evidence for additive effects of the combined intervention.

Contrary to our expectations and previous findings (9), the absence of additive effects suggests that combining brain stimulation with no-go inhibition training does not necessarily enhance intervention effects beyond cognitive training alone. This discrepancy between the earlier and current findings may be related to key methodological differences. Specifically, whereas the earlier study used a single food target (i.e., chocolate snacks) across all participants assessing actual in-lab food intake using a bogus taste test, the present study used individually tailored food targets (e.g., chocolate snacks, other snack foods, or bread) and assessed self-reported food craving and food choices for later consumption. We speculate that these individually tailored targets were more motivationally salient and contributed to responses based on pre-existing preferences. In particular, during the food choice task, participants’ decisions may have been influenced by consideration of later consumption, attenuating the sensitivity to the effects of high-frequency rTMS and its additional contribution. Furthermore, previous research revealed that prefrontal brain stimulation reduced craving for sweet or carbohydrate-rich foods, but not for savory or high-fat foods (62, 63). These findings suggest that prefrontal brain stimulation effects may vary across food types, potentially reflecting differences in underlying reward-related neural circuits engaged by distinct food categories (64).

Despite the absence of rTMS intervention, no-go inhibition training demonstrated consistent effects across outcome measures. Specifically, participants who consistently withheld responses to tempting high-calorie food showed decreased positive implicit attitudes toward high-calorie foods and a significant increase in explicit craving for low-calorie foods following training. They also made healthier food choices than those who responded and withheld responses with equal frequency. Notably, however, no-go training-dependent changes were not observed in explicit craving for high-calorie foods, despite these foods being the specific targets of the training. Because participants had preexisting preferences for high-calorie target foods, these preferences, together with the self-reported nature of craving ratings, may have limited our ability to detect changes in craving for these foods. In this context, the training effect may have been more evidently expressed as a training-dependent shift toward low-calorie foods, which also led to healthier (low-calorie) food choices during the food choice task. The greater increase in positive affect in the no-go training group, relative to the neutral training group, may have supported the expression of this shift and may be related to improved emotion regulation, consistent with participants’ successful no-go inhibition during training.

Our moderation analysis results indicated the importance of preexisting preferences for high-calorie foods in the training effect. Particularly, no-go training effects on food-choice behavior were more pronounced among participants with relatively low to moderate preferences for high-calorie foods, whereas those with strong preferences did not show a significant training effect. This suggests that the strength of preexisting preference may influence responsiveness to inhibition training. In addition, exploratory moderation analyses also indicated that menstrual phase moderated no-go training effects on food choice, such that significant training effects were found among women in the luteal phase but not in non-luteal phases. Given that the luteal phase is associated with elevated appetite, food intake, and food cue reactivity (65, 66), increased sensitivity to food cues may increase the likelihood of training-dependent changes. This view is consistent with previous research showing stronger inhibition training effects among individuals with relatively high appetite or more frequent food intake (67, 68). These moderator results suggest the distinct contributions of static and dynamic individual difference factors, warranting further investigation in follow-up research.

Our main finding regarding the effects of no-go inhibition training is consistent with our previous work (9) and extends previous findings in several ways. First, we demonstrated that comparable effects can be achieved using VR-based no-go training. Second, we showed that VR-based no-go training dependent changes were evident in both explicit measures of craving and food choice and an implicit measure of positive attitude. In addition, moderation analyses suggested that these training effects may depend on individual differences in baseline characteristics, such as preexisting preferences and menstrual cycle phases.

Overall, the current findings support that simple association-based learning to inhibit motor actions to tempting foods can change individuals’ responses to these foods. To explain the mechanism underlying these changes, the BSI theory suggests that repeated motor inhibition results in devaluation of tempting foods in order to resolve a conflict between strong response tendencies to tempting foods and task requirements (8, 32). This account is supported by the observed training-dependent decrease of positive attitudes toward high-calorie foods in the no-go training group. Although the potential efficacies of inhibitory control training have been actively examined in the literature, its application to clinical populations remains limited (5, 6, 33). Recently, a meta-analytic study examined the efficacy of inhibition training across multiple unhealthy behaviors, including smoking, drinking and unhealthy eating, in clinical and at-risk populations (69). Results revealed that training-dependent effect size was not significant, suggesting a caution in using inhibition training as a stand-alone intervention to treat clinical symptoms. These findings suggest the need for further research to determine whether inhibitory control training, when combined with other interventions, such as psychological or neuromodulatory methods, can enhance its effectiveness in clinical populations.

The current study has several limitations that warrant further consideration. First, no measurable effects of rTMS were observed across outcome measures used in the current study. Possibly, a single session of brain stimulation may have been insufficient to elicit detectable changes (70), as meta-analytic findings indicate that greater stimulation dosing (e.g., number of sessions or stimulation pulses) is associated with larger modulatory effects (71–73). In addition, although a fixed stimulation intensity was employed based on recent empirical findings (40–42) and established protocols in our laboratories (9, 74), this method might not have been optimal for all participants, thereby reducing the sensitivity to detectable effects. Also, we employed an off-line rTMS, in which brain stimulation preceded the no-go training. Given the growing recognition that brain stimulation effects are state-dependent (75–77), applying rTMS concurrently with the no-go task may have effectively facilitated the neural circuits underlying inhibitory control. However, the evidence regarding the efficacy of online stimulation is also mixed, and thus its contribution to the present results is unclear (42).

Second, our sample included only healthy women, which can limit the generalizability of the findings. Future studies should examine whether the findings extend to men and clinical populations, such as individuals with obesity or eating disorders. Third, the persistence of VR-based no-go training remains unknown as the present study assessed only immediate effects following training. An earlier study suggests that the effects of single-session no-go training can persist for up to one week (78).

Despite these limitations, the current study demonstrates that VR-based no-go training can robustly influence food-related responses, even in the absence of rTMS effects. Our findings suggest the promising potential of VR-based no-go training for shaping food-related responses, while highlighting the importance of considering individual differences in future research.

## 5 Conclusion

Building on our previous study, the current study aimed to examine the individual and combined effects of VR-based no-go training and rTMS on implicit attitudes, explicit craving and food choice. The results showed that VR-based no-go training reliably modified these outcome measures. These effects were independent of rTMS, with no evidence of additional benefits. Although no measurable effects of rTMS were observed, this does not rule out potential neuromodulatory contributions, as the use of single-session stimulation and the choice of stimulation-related parameters may not have been optimal to elicit detectable effects. Importantly, the consistent effects of no-go training across our previous and current studies suggest that inhibitory control training offers a worthwhile direction for further investigation in clinical context. Future research should determine whether combining inhibitory control training with neuromodulation enhances its effectiveness by optimizing stimulation conditions, assessing long-term effects, and extend findings to broader and clinical populations.

## Data Availability

The raw data supporting the conclusions of this article will be made available by the authors, without undue reservation.
