# Supplementary material for: Virtual reality-based inhibition training influences food-related responses: no additional effects of repetitive transcranial magnetic stimulation
Source: Front Psychiatry. 2026 May 8;17:1801985. doi: 10.3389/fpsyt.2026.1801985 (PMC13194486; doi:10.3389/fpsyt.2026.1801985)
Supplement: Supplementary file 1 [file Supplementaryfile1.docx]

Supplementary Material

# Supplementary Tables

**Supplementary Table S1.** The General Preference Rating Scale

The General Preference Rating Scale consisted of 13 everyday items, including five food items representing distinct categories (bread, chocolate, snack foods, fruits, and vegetables). Participants rated their preference for each item using a 9-point Likert scale.

Instructions: Please rate your preference for the following words.

| 1. Social media | 1 ------ 2 ------ 3 ------ 4 ------ 5 ------ 6 ------ 7 ------ 8 ------ 9 | | |
| --- | --- | --- | --- |
|  | Not at all | Moderately | Strongly |
| 2. Chocolate | 1 ------ 2 ------ 3 ------ 4 ------ 5 ------ 6 ------ 7 ------ 8 ------ 9 | | |
|  | Not at all | Moderately | Strongly |
| 3. Alcohol | 1 ------ 2 ------ 3 ------ 4 ------ 5 ------ 6 ------ 7 ------ 8 ------ 9 | | |
|  | Not at all | Moderately | Strongly |
| 4. TV show | 1 ------ 2 ------ 3 ------ 4 ------ 5 ------ 6 ------ 7 ------ 8 ------ 9 | | |
|  | Not at all | Moderately | Strongly |
| 5. Video game | 1 ------ 2 ------ 3 ------ 4 ------ 5 ------ 6 ------ 7 ------ 8 ------ 9 | | |
|  | Not at all | Moderately | Strongly |
| 6. Snack foods | 1 ------ 2 ------ 3 ------ 4 ------ 5 ------ 6 ------ 7 ------ 8 ------ 9 | | |
|  | Not at all | Moderately | Strongly |
| 7. Reading | 1 ------ 2 ------ 3 ------ 4 ------ 5 ------ 6 ------ 7 ------ 8 ------ 9 | | |
|  | Not at all | Moderately | Strongly |
| 8. Vegetables | 1 ------ 2 ------ 3 ------ 4 ------ 5 ------ 6 ------ 7 ------ 8 ------ 9 | | |
|  | Not at all | Moderately | Strongly |
| 9. Cosmetics | 1 ------ 2 ------ 3 ------ 4 ------ 5 ------ 6 ------ 7 ------ 8 ------ 9 | | |
|  | Not at all | Moderately | Strongly |
| 10. Social gathering | 1 ------ 2 ------ 3 ------ 4 ------ 5 ------ 6 ------ 7 ------ 8 ------ 9 | | |
|  | Not at all | Moderately | Strongly |
| 11. Fruits | 1 ------ 2 ------ 3 ------ 4 ------ 5 ------ 6 ------ 7 ------ 8 ------ 9 | | |
|  | Not at all | Moderately | Strongly |
| 12. Shopping | 1 ------ 2 ------ 3 ------ 4 ------ 5 ------ 6 ------ 7 ------ 8 ------ 9 | | |
|  | Not at all | Moderately | Strongly |
| 13. Bread | 1 ------ 2 ------ 3 ------ 4 ------ 5 ------ 6 ------ 7 ------ 8 ------ 9 | | |
|  | Not at all | Moderately | Strongly |

**Supplementary Table S2.** Distribution of food categories selected as training targets within each group

|  | Active/No-go  (n=29) | Active/Neutral  (n=30) | Sham/No-go  (n=30) | Sham/Neutral  (n=29) |
| --- | --- | --- | --- | --- |
| Bread | 12 | 14 | 14 | 12 |
| Chocolate | 10 | 12 | 9 | 10 |
| Snacks | 7 | 4 | 7 | 7 |

Note. Training targets were assigned based on participants’ highest baseline preference ratings. Values represent participant counts.

**Supplementary Table S3.** Attribute words used in the Single Category Implicit Association Test (SCIAT)

| Attribute Category | Word | Frequency | Valence | Arousal | Concreteness |
| --- | --- | --- | --- | --- | --- |
| Positive | Joy | 1268 | 8.24 | 5.82 | 2.87 |
|  | Satisfaction | 363 | 7.59 | 3.37 | 2.91 |
|  | Refreshment | 188 | 8.01 | 4.25 | 2.71 |
|  | Liking | 276 | 7.46 | 4.49 | 2.94 |
|  | Pleasure | 691 | 7.73 | 5.88 | 3.04 |
|  | Fantastic | 320 | 7.44 | 5.85 | 2.13 |
|  | **Mean** | **517.67** | **7.75** | **4.94** | **2.77** |
| Negative | Discomfort | 462 | 2.78 | 6.47 | 3.07 |
|  | Dissatisfaction | 1262 | 2.88 | 6.96 | 3.05 |
|  | Disappointment | 324 | 2.70 | 4.95 | 2.76 |
|  | Regret | 255 | 2.93 | 5.23 | 2.87 |
|  | Rejection | 170 | 2.75 | 5.97 | 3.02 |
|  | Indifference | 260 | 2.33 | 4.07 | 3.13 |
|  | **Mean** | **455.50** | **2.73** | **5.61** | **2.98** |

Note. Data source: Hong Y, Nam Y-e, Lee Y. Developing Korean affect word list and it's application. *Korean J Cogn Sci*. (2016) 27(3):377-406. doi: 10.19066/cogsci.2016.27.3.002

## Supplementary Figures

| **High-calorie foods** | | | | | | | |
| --- | --- | --- | --- | --- | --- | --- | --- |
| *Bread* | | | | | | | |
| **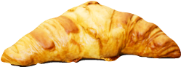** | **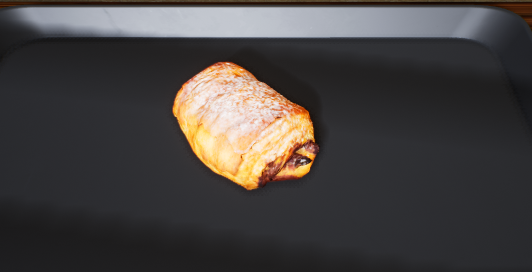** | **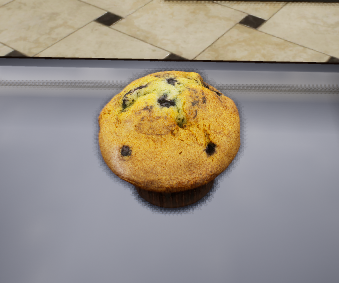** | **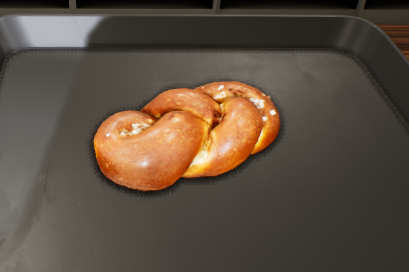** | **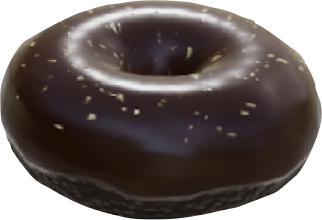** | **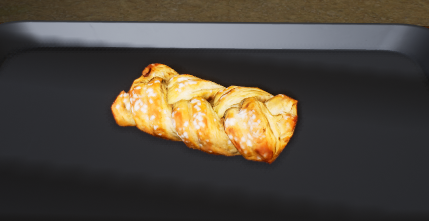** | **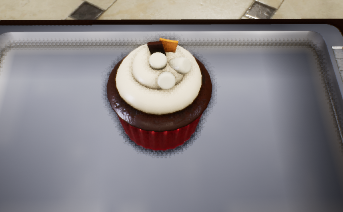** | **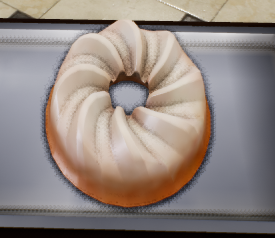** |
| *Chocolate* | | | | | | | |
| **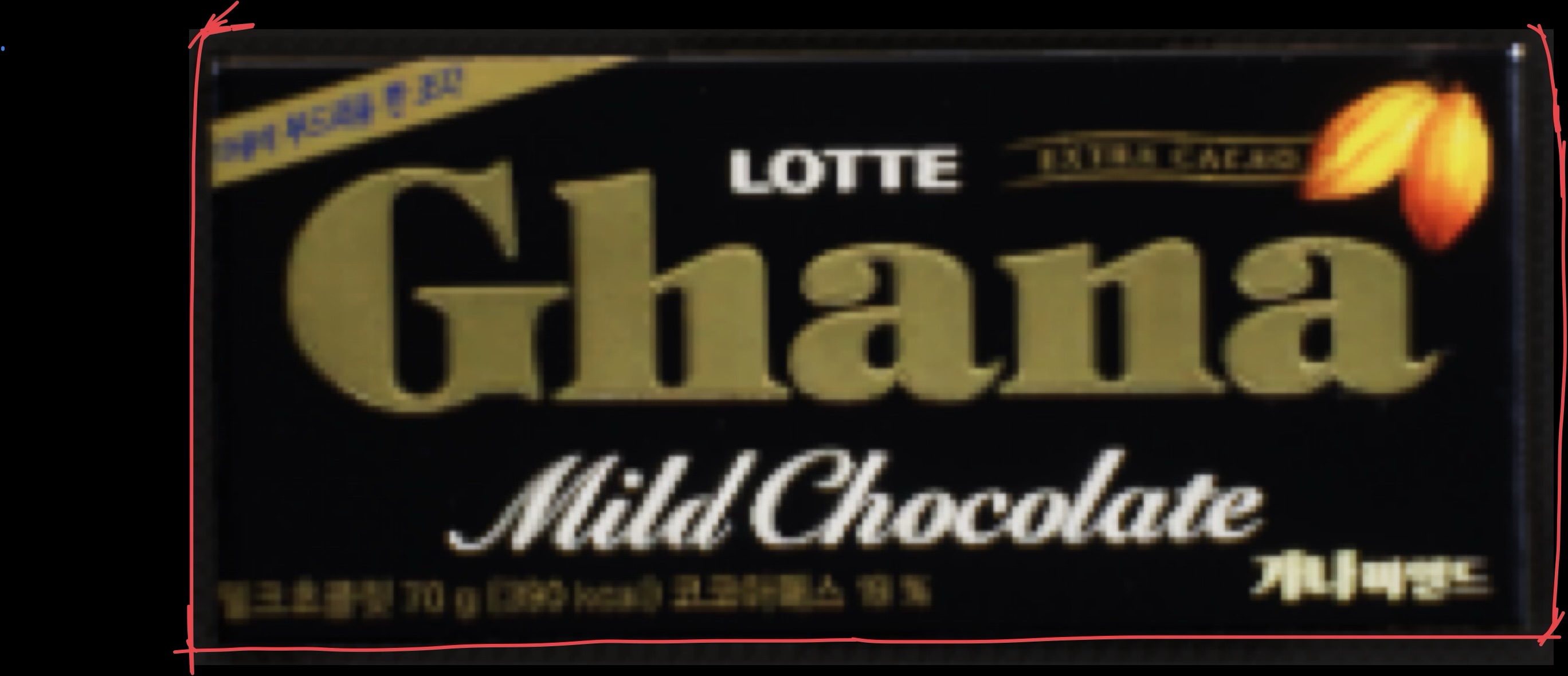** | **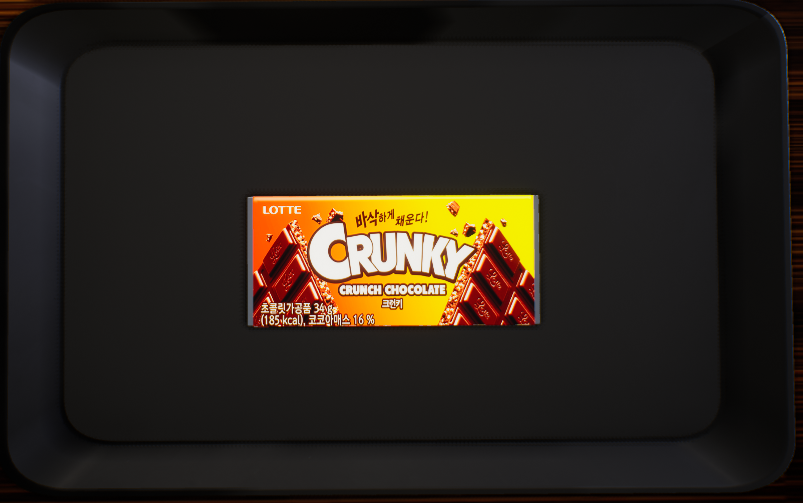** | **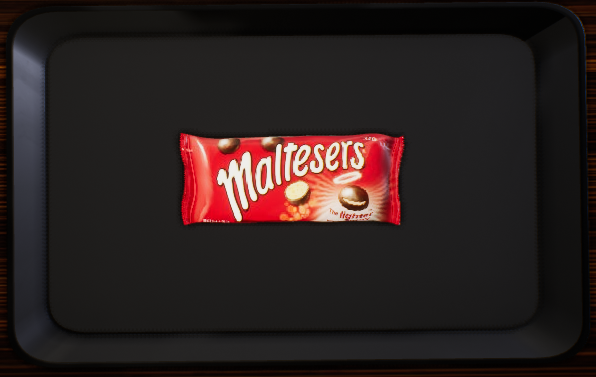** | **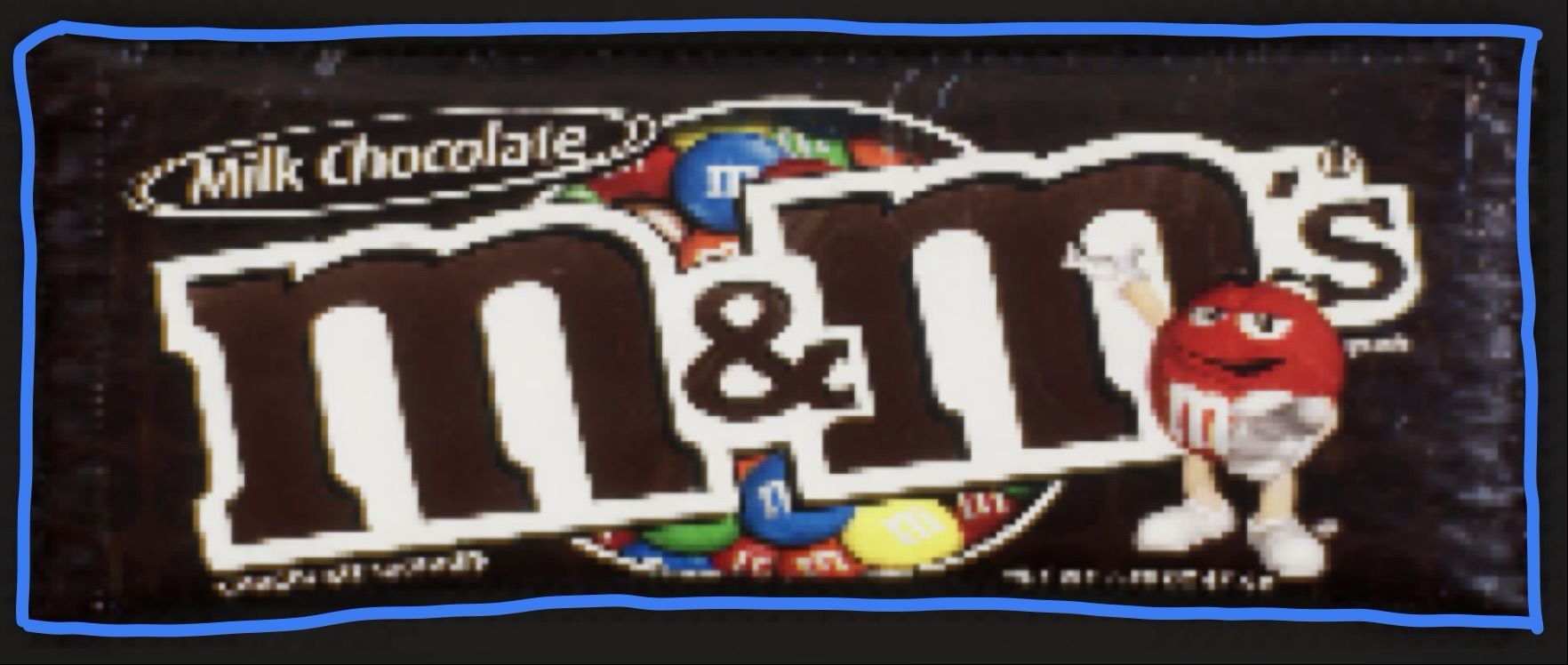** | **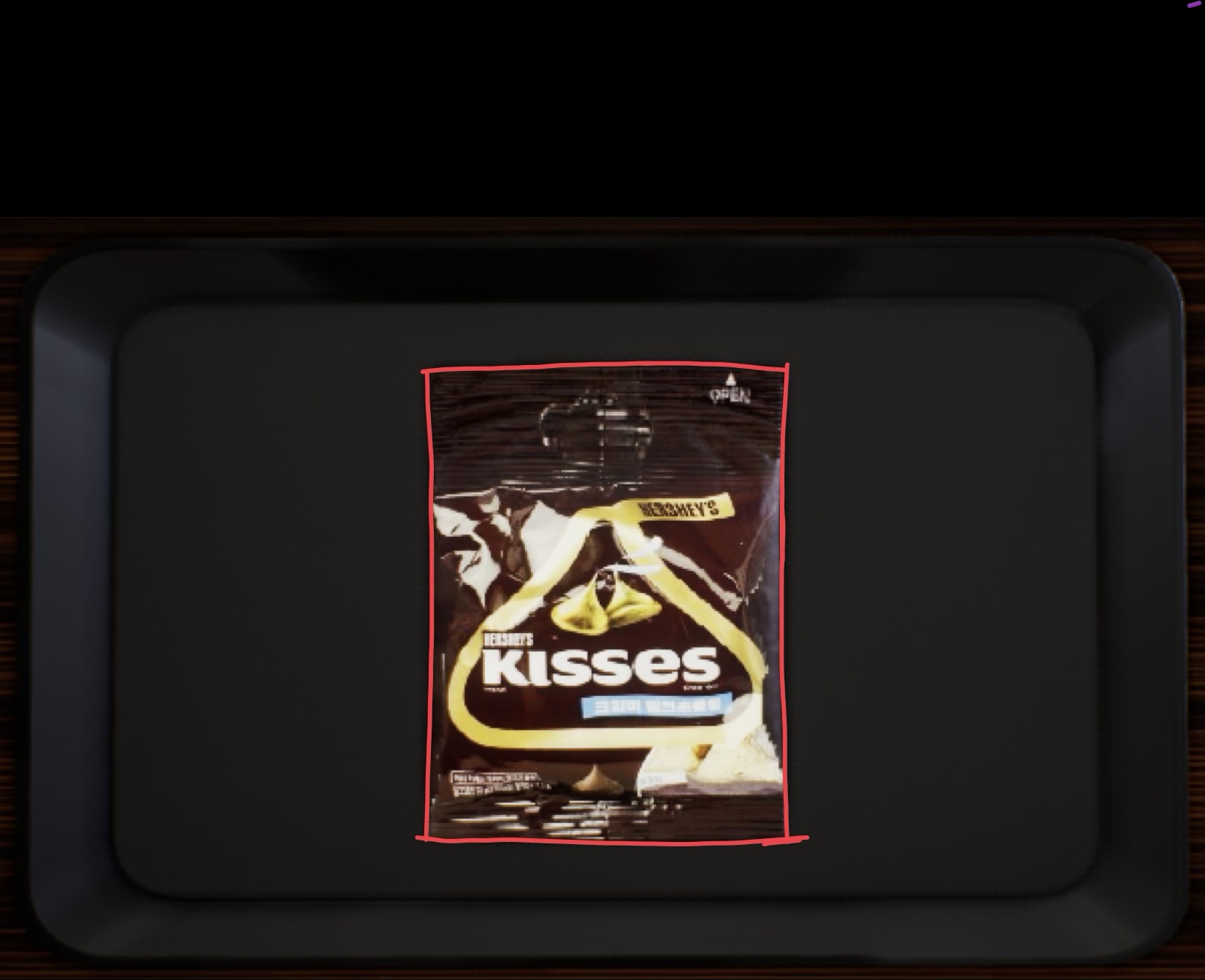** | **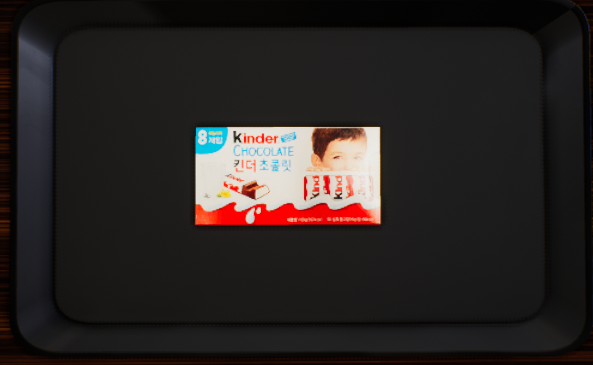** | **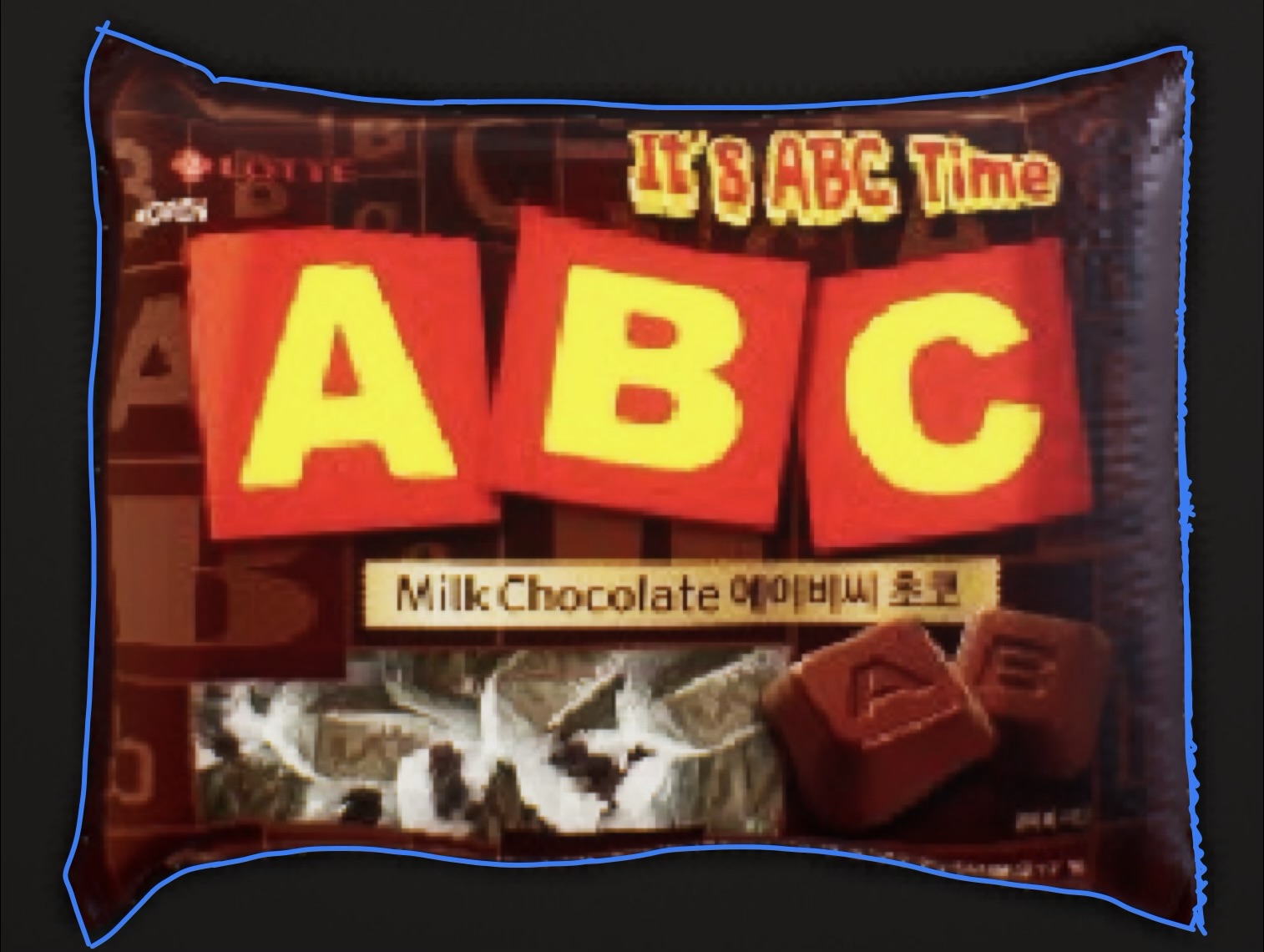** | **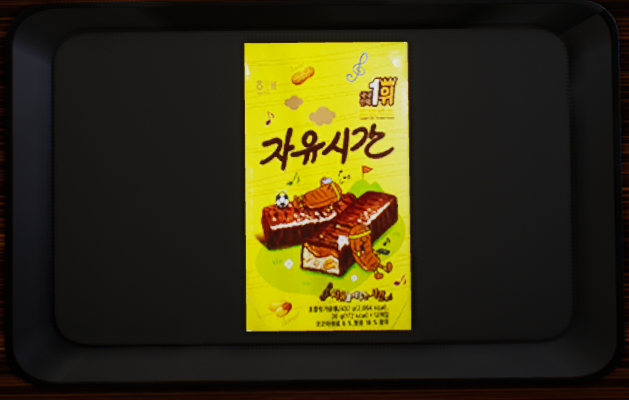** |
| *Snack* | | | | | | | |
| **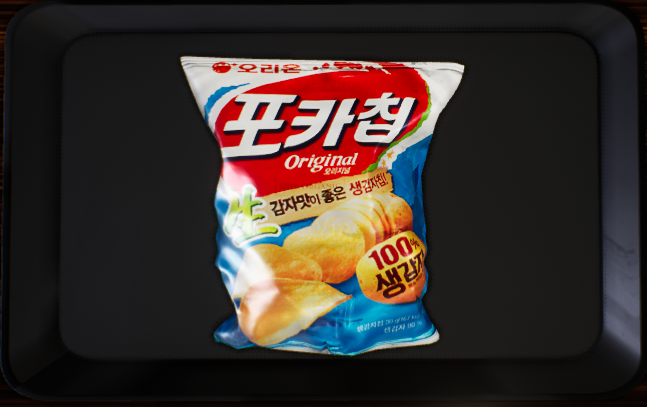** | **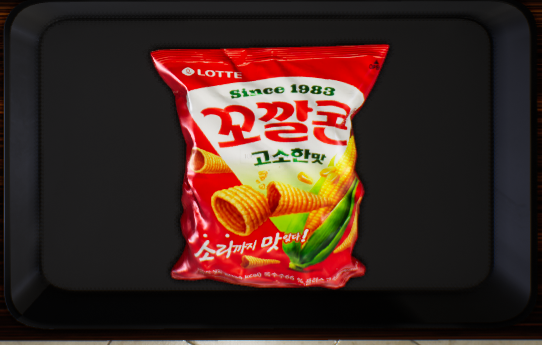** | **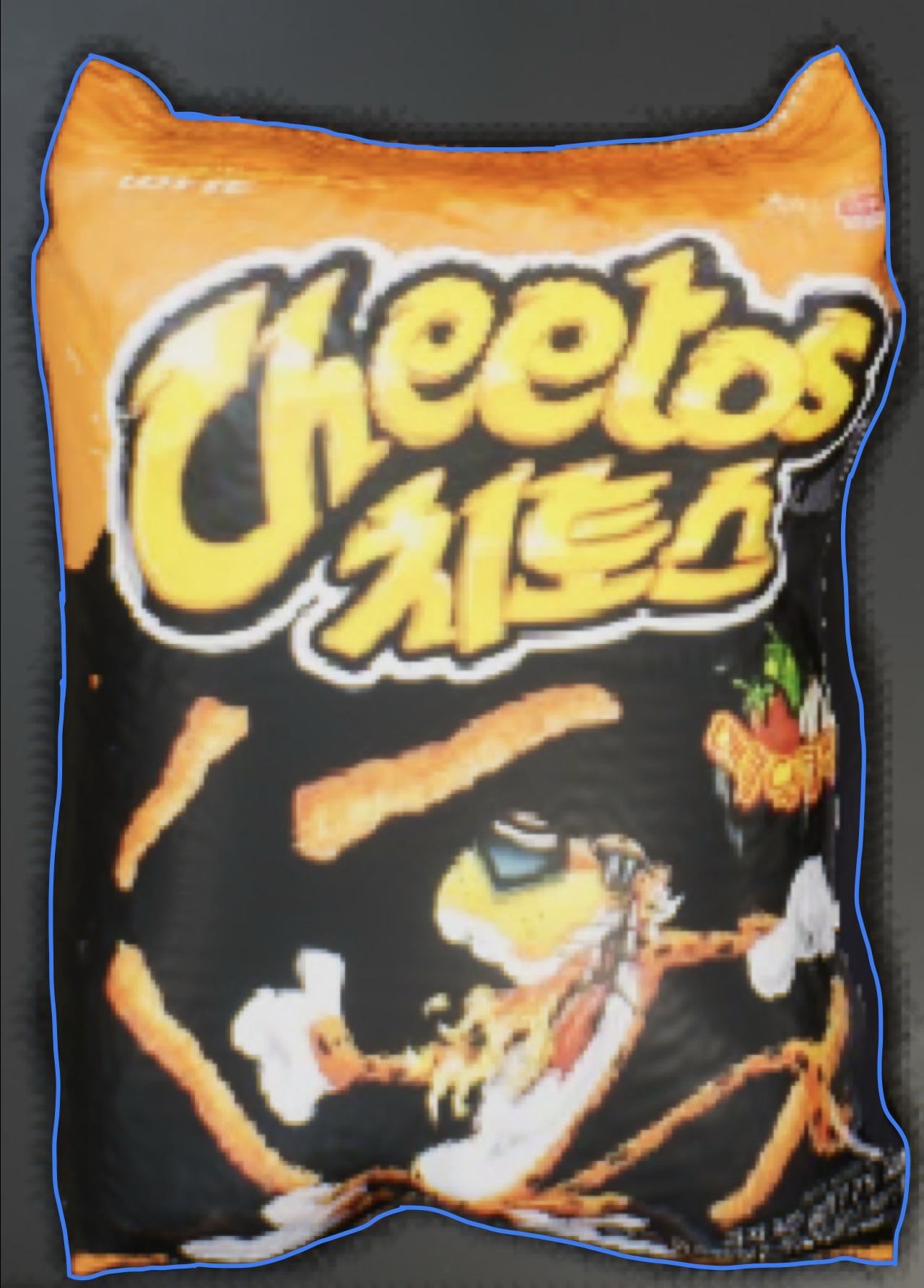** | **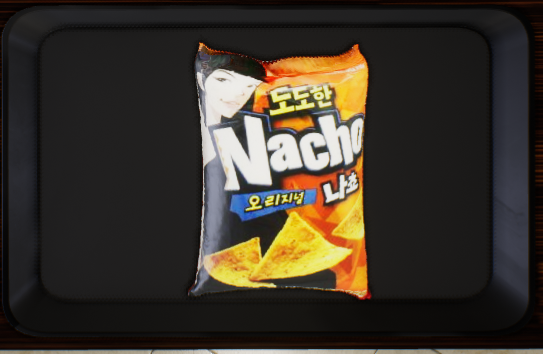** | **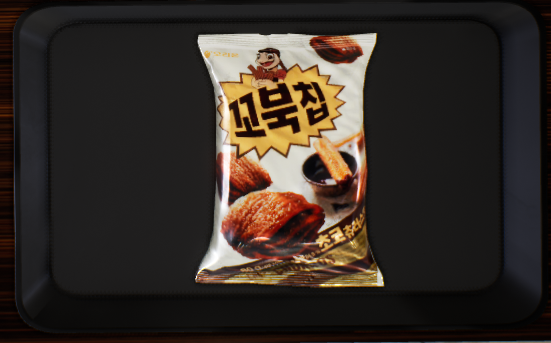** | **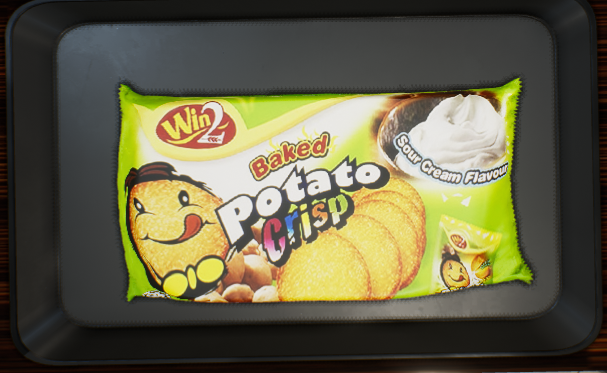** | **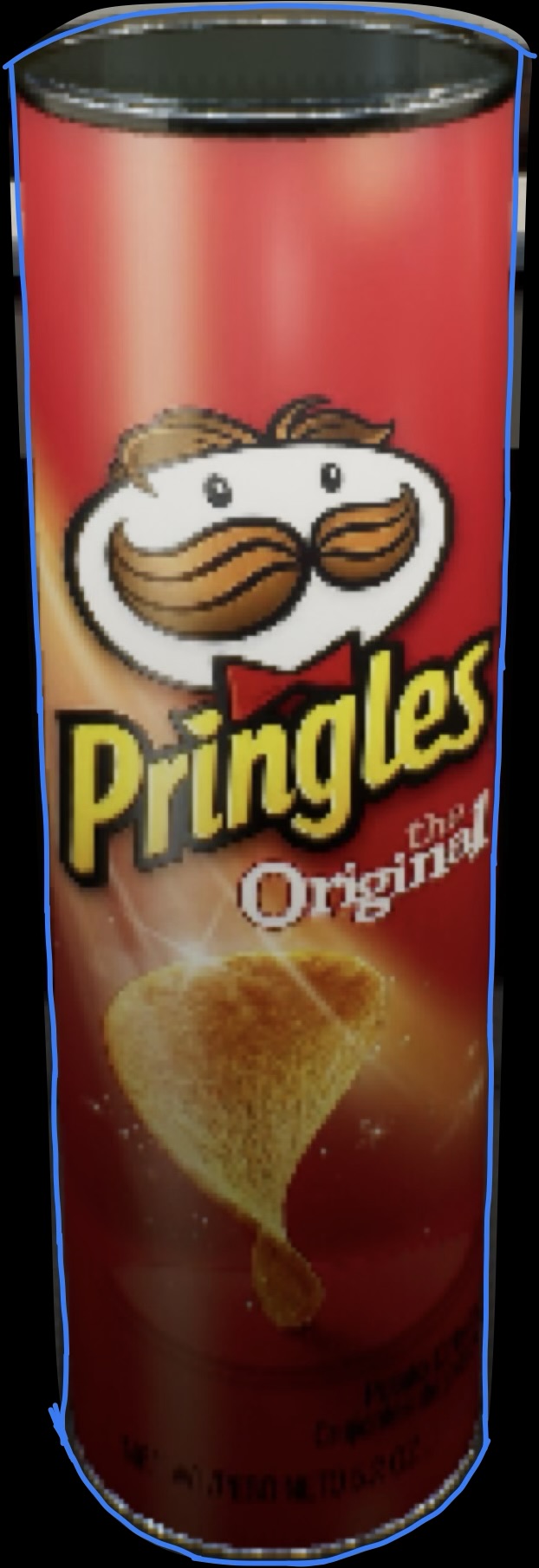** | **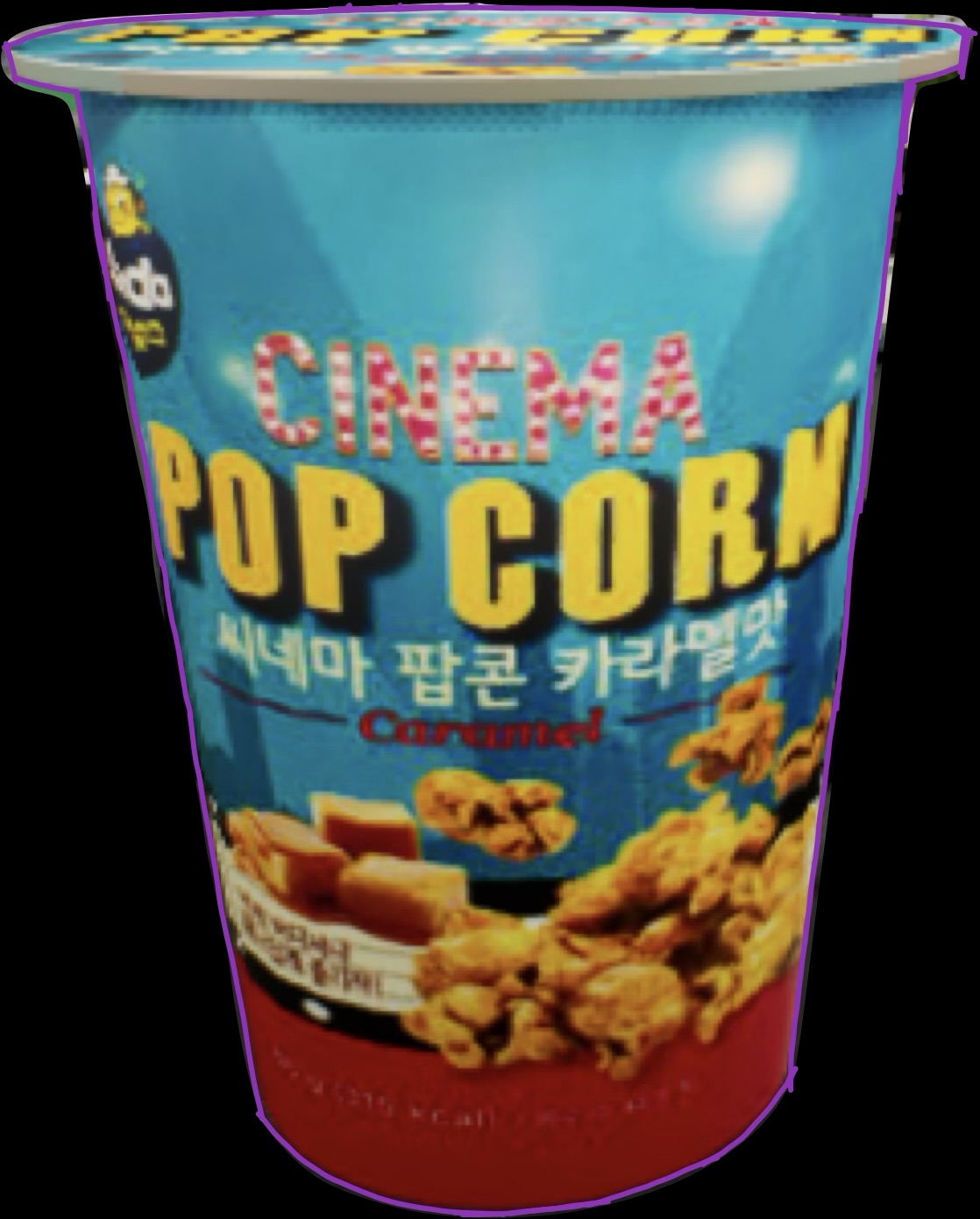** |
| **Low-calorie foods** | | | | | | | |
| **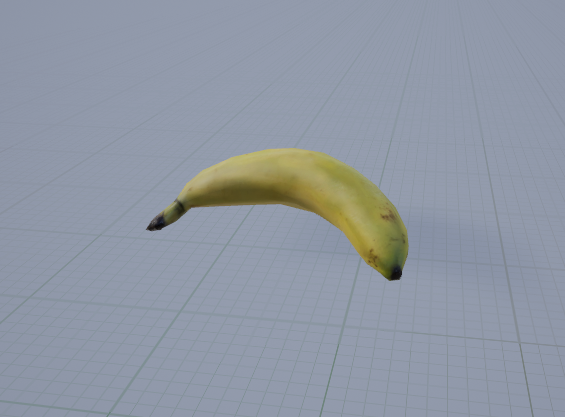** | **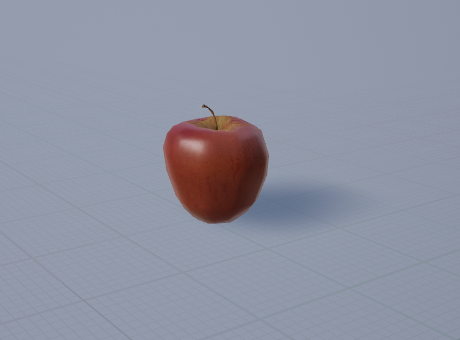** | **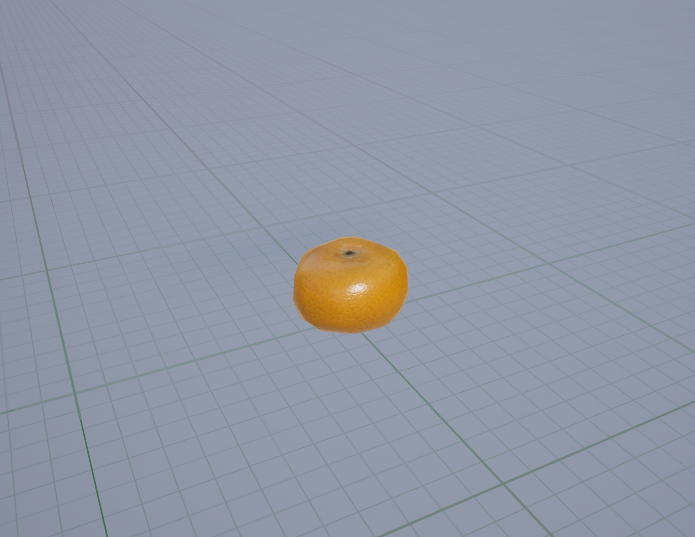** | **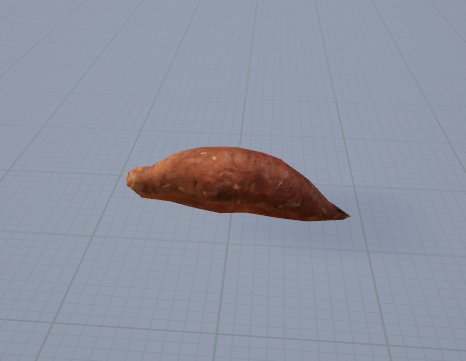** | **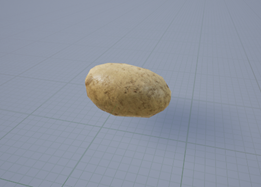** | **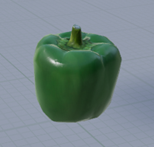** | **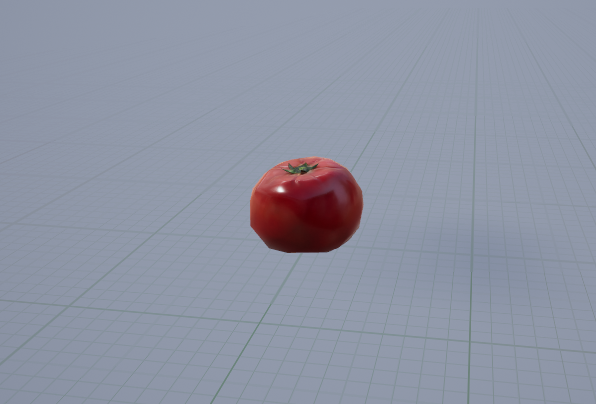** | **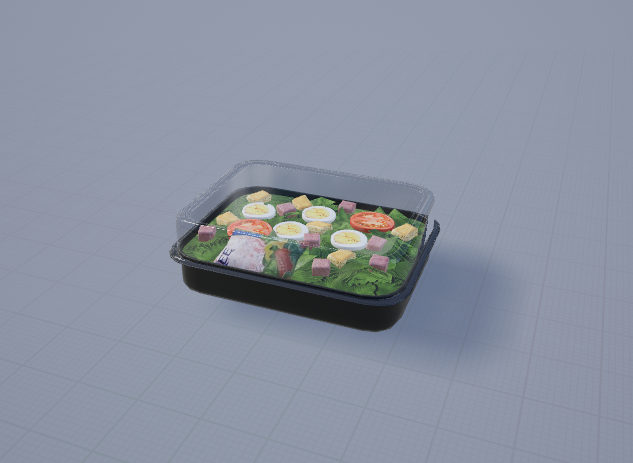** |
| **Everyday objects** | | | | | | | |
| **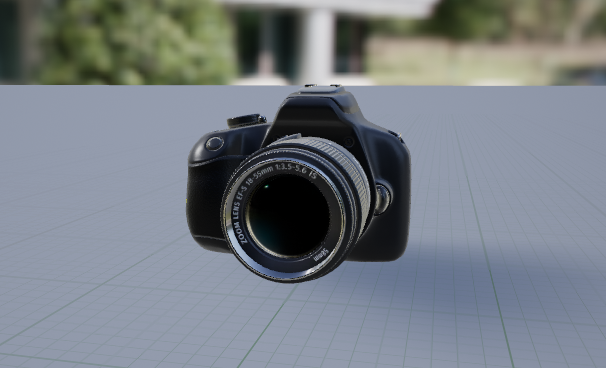** | **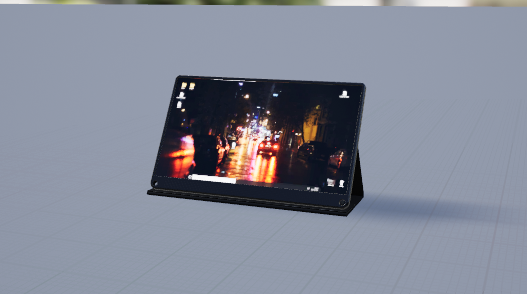** | **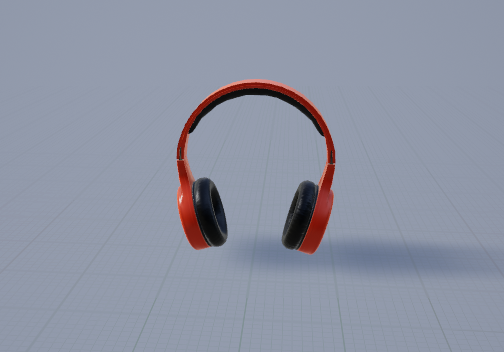** | **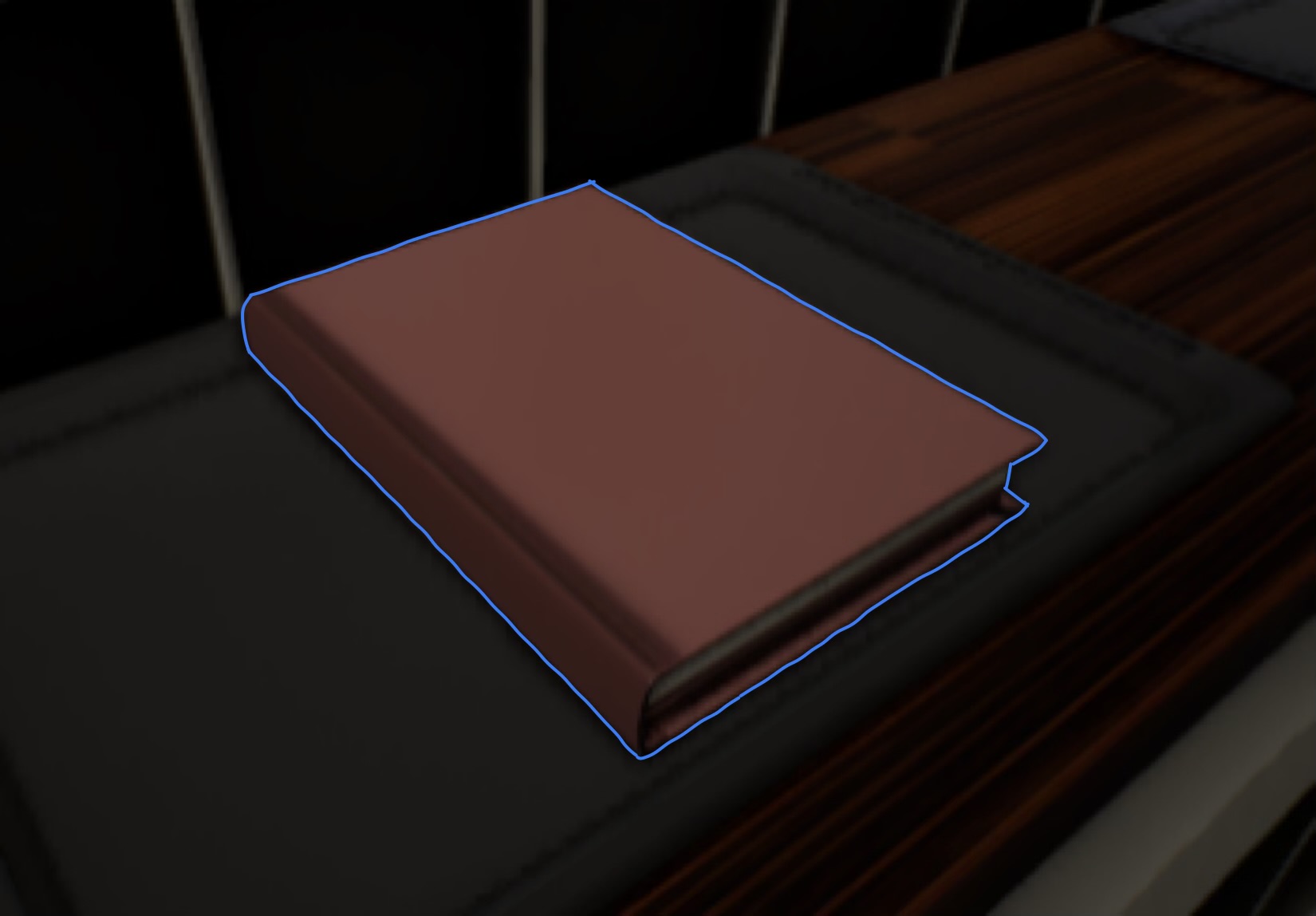** | **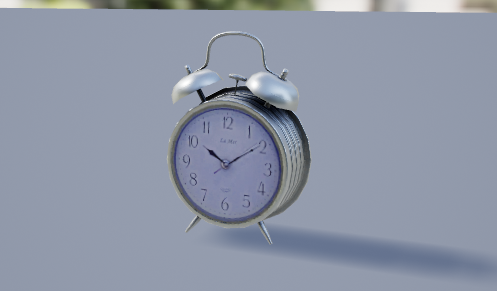** | **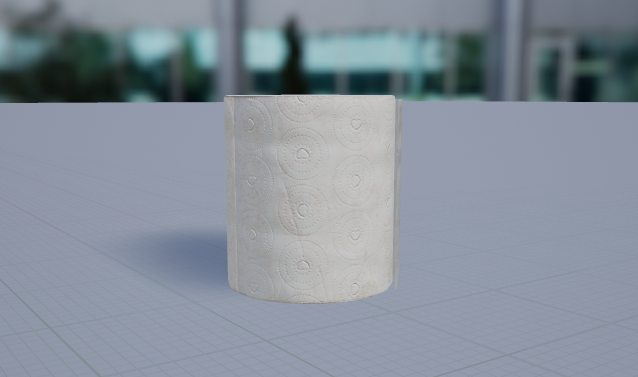** | **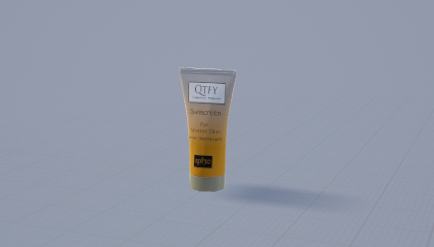** | **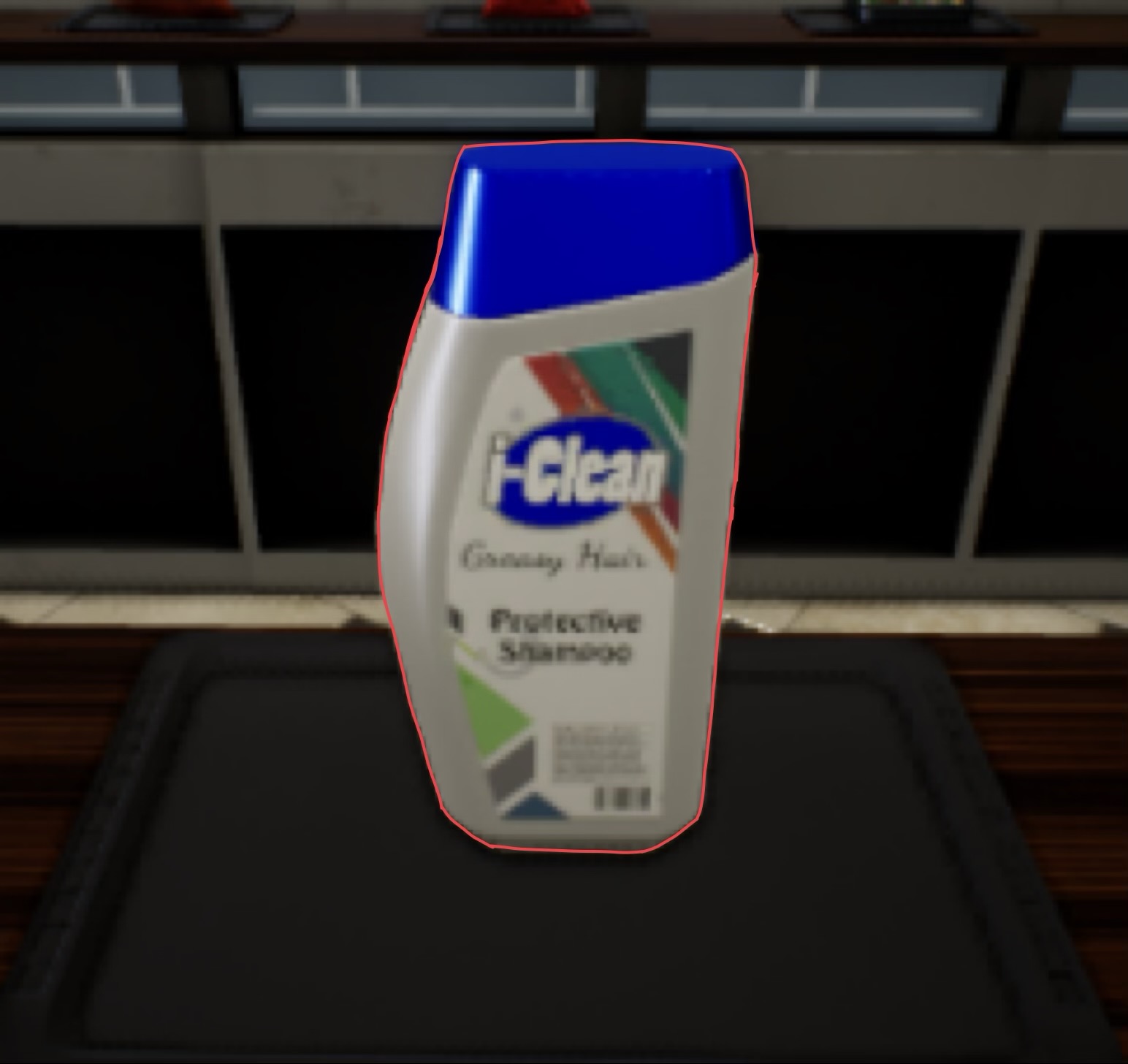** |

**Supplementary Figure S1.** 3D stimuli used in the virtual reality no-go training


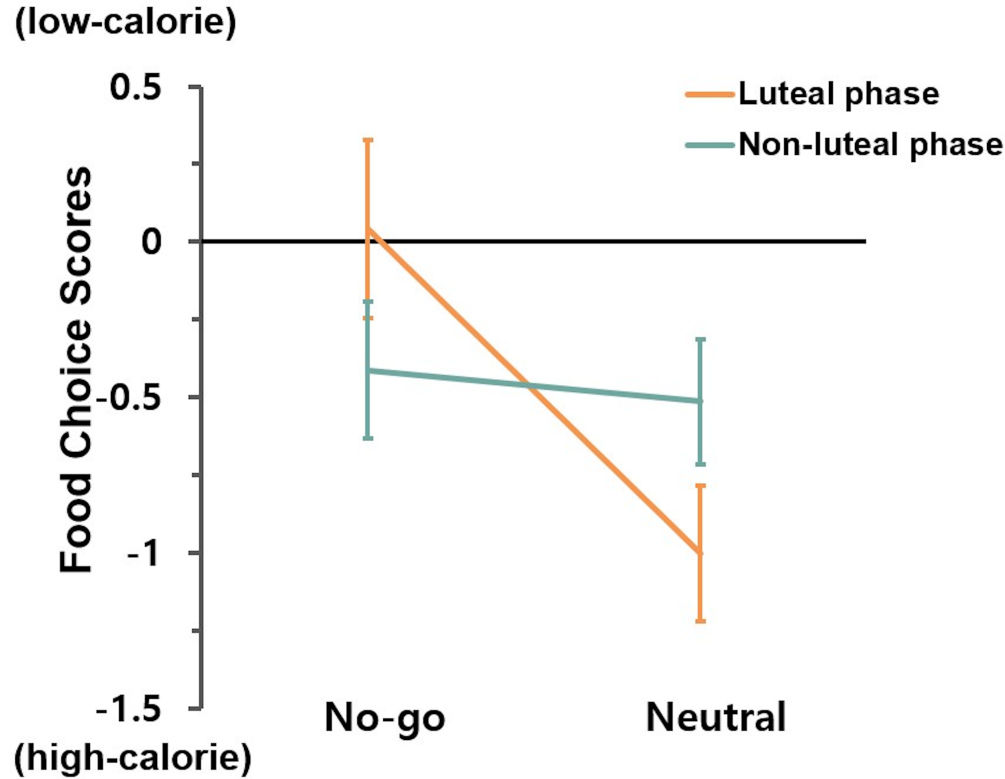


**Supplementary Figure S2.** Moderating effect of menstrual cycle phase on food choice
